# Pan-cancer predictive survival model development and evaluation using electronic health record and genetic data across 10 cancer types

**DOI:** 10.1007/s12672-025-02523-1

**Published:** 2025-05-12

**Authors:** Jurgita Gammall, Alvina G. Lai

**Affiliations:** 1 Institute of Health Informatics, University College London, 222 Euston Road, London, NW1 2DA UK; 2 Oracle Global Services Limited, London, UK

**Keywords:** Cancer, Prognosis, Survival, Predictive model, Machine learning, Genetics, Electronic health records

## Abstract

The growing burden of cancer and recent surge in healthcare data availability call for new ways of analysing this multifactorial disease and improving patient outcomes. The aim of this study is to develop and evaluate prognostic cancer survival models across ten common cancer types based on a large patient sample. We compare the performance of different machine learning algorithms and assess the added value of genetic information in cancer prognosis. We also provide ways to improve model explainabilty which is critical for model adoption in clinical practice. This study included data from 9977 patients with bladder, breast, colorectal, endometrial, glioma, leukaemia, lung, ovarian, prostate, and renal cancers. Genetic data collected through the 100,000 Genomes Project was linked with clinical and demographic data provided by the National Cancer Registration and Analysis Service, Hospital Episode Statistics and Office for National Statistics. More than 500 prognostic features were assessed and four machine learning algorithms including Elastic Net Cox proportional hazards regression, random survival forest, gradient boosting survival and DeepSurv neural network were developed in this study. Most models achieved good performance varying from 60% in bladder cancer to 80% in glioma with the average C-index of 72% across all cancer types. Different machine learning methods achieved similar performance with DeepSurv model slightly underperforming compared to other methods. Addition of genetic data improved performance in endometrial, glioma, ovarian and prostate cancers, showing its potential importance for cancer prognosis. Patient’s age, stage, grade, referral route, waiting times, pre-existing conditions, previous hospital utilisation, tumour mutational burden and mutations in gene TP53 were among the most important features in cancer survival modelling. By offering a comprehensive set of predictive models for cancer survival, this study fills a critical gap in our understanding of cancer prognosis and provides new tools for informing cancer treatment and consequently improving patient outcomes.

**Supplementary Information:**

The online version contains supplementary material available at 10.1007/s12672-025-02523-1.

## Introduction

The burden of cancer has been growing worldwide with over 35 million new cancer cases predicted in 2050 [1], calling for more research efforts to advance the understanding of the disease and improve patient outcomes. Cancer is a complex disease, influenced by a variety of factors including genetics, demographics, biology, environment and lifestyle. Recent surge in data availability through routinely used electronic health record systems and advances in genetic data collection enable new ways of analysing this multifactorial disease such as predictive model development.

There is a considerable number of cancer prognostic models developed over the years, showing that data-driven approaches can improve the accuracy of cancer survival prediction when compared to predictions based on experience of clinicians or cancer staging and grading alone [2–4]. In a previous systematic review we conducted [5], we assessed the published evidence on clinicopathological and genetic factors associated with cancer prognosis. We found that the vast majority of previous cancer studies used statistical methods such as Cox proportional hazards regression to model cancer prognosis. A known limitation of traditional statistical methods such as regression is that these methods often do not work well on high-dimensional data and for problems with non-linear relationships. The more sophisticated design of machine learning algorithms allows handling high-dimensional data and complex relationships between prognostic factors and target outcome [6] and a significant rise in the use of machine learning models has been observed in the recent years [7]. Algorithms using state of the art techniques such as ensemble trees and deep learning models were developed to predict cancer survival and have shown to outperform the more traditional regression-based methods [8–12]. However, some models are limited by a small sample size or lack in validation methods and interpretability [13].

Prognostic cancer survival models developed in this study were based on a large patient sample and were validated using cross validation techniques as well as a set-aside test sample. Four different machine learning algorithms were selected for development of predictive cancer survival models, namely Cox Elastic Net regression model (CoxNet), Random Survival Forest (RSF), Gradient Boosting Survival (GBS) model and DeepSurv Neural Network (DeepSurv) [14]. CoxNet model was chosen to represent more traditional regression-based methods in cancer survival modelling, as it was the most popular method observed in previous literature [5]. Ensemble tree methods are known to perform well in modelling healthcare outcomes with random forest and gradient boosting approaches proven to be more effective than single decision tree methods [13]. DeepSurv model was selected to represent the most complex deep learning approaches, which have increasingly been used in predictive modelling in the recent years [15]. Machine learning algorithms are known to be better at handling high-dimensional data compared to more traditional regression-based approaches and are able to consider complex relationships and interactions between different prognostic factors.

The data available through the 100,000 Genomes Project led by Genomics England [16] provides a unique opportunity to perform a multidimensional analysis on thousands of patients with different types of cancer by linking genetic, clinicopathological and demographic data. This study builds upon our previous work [17], which summarised the results from the analysis of prognostic factors utilising genetic data collected by Genomics England, linked with Hospital Episode Statistics (HES) [18] data, National Cancer Registration and Analysis Service (NCRAS) [19] data and Office of National Statistics (ONS) [20] data for a total of 9977 patients across 10 cancer types. In our previous study we identified hundreds of clinicopathological and genetic factors associated with cancer survival through using Cox proportional hazards regression adjusted for age, sex and stage. This study addresses the complex nature of cancer disease by taking into account interactions between hundreds of prognostic factors and modelling complicated non-linear relationships by using more advanced computational methods. In this study, we also test and assess different data pre-processing methods applied in machine learning model development, such as missing data imputation and feature scaling techniques.

The primary aim of this study is to develop and evaluate adept prognostic cancer survival models across ten common cancer types based on a large patient sample. Furthermore, we seek to assess the usefulness of different machine learning algorithms in cancer survival modelling by comparing their performance across ten cancer types and different subsets of data. Additionally, we aim to assess the added value of genetic information in cancer prognosis by comparing the results of models with and without genetic data. Finally, we seek to address the common issue of machine learning models being seen as “black box” algorithms by applying various model interpretability approaches. By offering a comprehensive set of predictive models for cancer survival, this study seeks to fill a critical gap in our understanding of cancer prognosis and aims to provide new tools for informing cancer treatment and consequently improving patient outcomes.

## Methods

### Data and patient cohort

Five data sources from Genomics England’s Main Programme v15 (available since 26 th May 2022) [21] were used for developing prognostic survival models, as described in our previous study [17]. Patient cohort was created using primary clinical data collected by Genomic Medicine Centres for participants upon enrolment in the 100,000 Genomes Project programme. The patient cohort consisted of 9,977 patients across ten cancer types, namely bladder, breast, colorectal, endometrial, glioma, leukaemia, lung, ovarian, prostate and renal cancers. This patient cohort was linked with NCRAS data for information about patient’s diagnosis, tumour and treatment, HES data for medical history and ONS data for mortality information. Genomics England’s Cancer Tiering data [22] was used to identify genetic information about patients, namely relevant single nucleotide variants.

Contrary to the analyses described in our previous study [17], we included all genes with mutations identified in our patient cohort. There were 18,965 genes with 3,675,866 variants identified in the pan-cancer cohort of 9,977 patients. Once filtered on pathogenic or likely pathogenic variants, 2,413 genes with 36,934 variants remained in the cohort. Additional filter was applied to the list of genes to remove genes with low frequencies in our cohort (fewer than 10 patients or less than 0.5% of all patients for each cancer type), which was not sufficient data for modelling. This resulted in 314 genes and 28,163 variants included in the study.

### Feature selection

The initial dataset contained more than 500 features including binary, categorical and numeric features across ten cancer types. Categorical features were converted to binary features using one-hot encoding and data was then split by cancer type. Categorical features were investigated by cancer type in order to identify categories with small patient count that are not suitable for modelling. Furthermore, features with high level of missing data (> 60% of patients) were removed from the data for each cancer type.

### Splitting data to train and test

Ten datasets were split into features (X) and target (y) subsets. Train and test datasets were created for each cancer type using 80% of the data for training and keeping 20% of the data for testing. five-fold cross validation was applied on training dataset during hyperparameter tuning. Table 1 provides a number of patients in train and test sets by cancer type. Full list of features considered for each cancer type, including their data source is provided in Supplementary File 1.

**Table 1:** Number of patients and count of features by cancer type

Cancer type	Number of patients	Number of patients in train set	Number of patients in test set	Total number of features	Number of numeric features	Number of genetic features
Bladder	353	282	71	99	13	12
Breast	2605	2084	521	179	19	30
Colorectal	2247	1797	450	426	15	288
Endometrial	762	609	153	238	14	139
Glioma	434	347	87	90	16	10
Leukaemia	238	190	48	77	13	6
Lung	1317	1053	264	149	15	18
Ovarian	436	348	88	98	13	10
Prostate	462	369	93	69	12	6
Renal	1123	898	225	132	15	9

### Cleaning outliers

All numeric features were capped at 99% percentile apart from the age feature. Percentiles were calculated for each feature and each cancer type to account for differences in cancer types. The age feature contained values from 18 to 96, which were valid and did not require outlier cleaning. Only upper bound capping was applied, because all features had a lower bound of 0 by definition.

### Missing data imputation

Three most popular missing data imputation methods, median imputation, K-nearest neighbour (KNN) imputation and multivariate feature imputation (Iterative Imputer) were tested for features with missing data in two cancer types: breast and colorectal. Breast cancer had the highest number of patients and numeric features. Colorectal cancer had the second highest number of patients. Different methods were assessed by comparing data distributions for each feature before and after imputation using box plots and histograms. Multivariate feature imputation with decision tree regressor performed best in most features. It was selected together with KNN imputation with 5 nearest neighbours as two best methods and tested in another cancer type, glioma. Glioma was chosen due to the second highest number of numeric features. Multivariate feature imputation with decision tree regressor performed best in glioma, and this method was selected as the final method for missing data imputation in all cancer types. Comparison of different missing data imputation methods was conducted on training dataset only. Multivariate feature imputation with decision tree regressor was fit on the training dataset and then used to transform both training and testing datasets.

### Feature scaling

Some machine learning methods such as artificial neural networks are sensitive to relative magnitude of features and therefore require feature scaling to be applied to the training data before the models can be trained. Min–max scaler was applied to all numeric features in the data. Min–max scaling is a standard feature scaling method, which preserves the shape of the original data distribution. Another reason why this approach was selected was the high number of binary features in the data. Min–max scaler transformed all numeric features to the same range as binary features (0 to 1) without needing to make changes to binary features.

Minimum and maximum values used for feature scaling transformation were calculated for each feature and cancer type using training subset of data. The calculated values from training data were then used to scale features in both training and testing datasets. Feature scaling can be applied but is not required for regression and decision tree-based machine learning algorithms, therefore multiple versions of training and testing datasets with and without feature scaling were created to assess the added value of feature scaling. Results from decision tree-based methods with feature scaling applied were also more comparable to the results using neural networks.

Although it is worth assessing whether feature scaling adds value when predicting cancer survival, machine learning models that require extensive data pre-processing and transformations may be less preferrable for real-world implementation due to multiple reasons. Firstly, additional data pre-processing tasks might be computationally expensive and require more time to produce the results, which could be an issue if predictions are used real time in a clinical setting. Secondly, data transformations reduce model interpretability and could negatively impact model’s adoption in clinical practice.

**Fig. 1:**
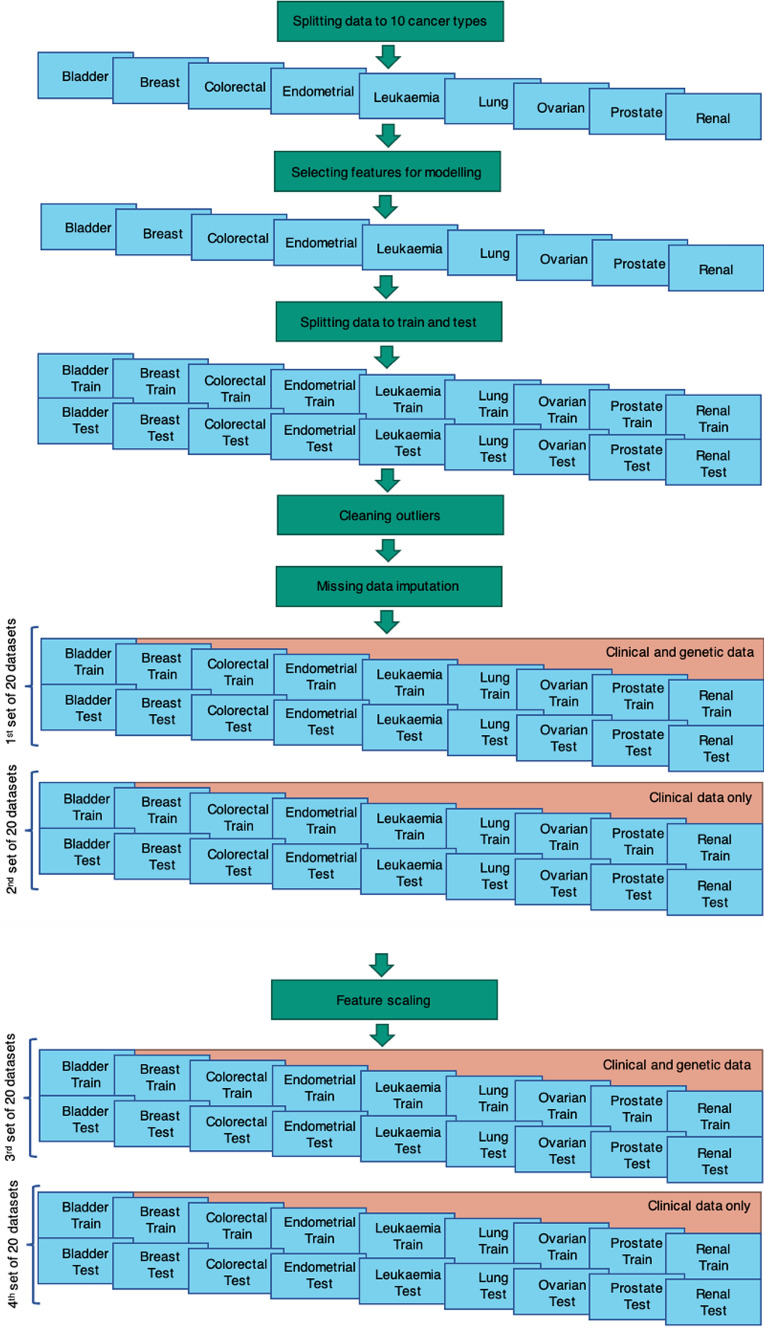
Summary of pre-processing steps and preparation of 80 datasets

Figure 1 provides a summary of pre-processing steps and preparation of the total of 80 datasets used for survival model development. Firstly, the data was split into 10 datasets by cancer type. Secondly, appropriate features were selected for each cancer type. Thirdly, each dataset was split into train and test subsets creating a total of 20 subsets of data. Cleaning of outliers and missing data imputation were then conducted on all 20 subsets of data. Using 20 subsets of data, two versions were created with and without genetic data leading to 40 datasets (see 1 st and 2nd sets in Fig. 1). Finally, a version of the same datasets with feature scaling applied was created, which led into additional 40 datasets (see 3rd and 4 th sets in Fig. 1).

### Machine learning algorithms

Machine learning survival models can handle censored data and take into account time-to-event information, therefore are applicable for predictive modelling of cancer survival. Survival time in years was used for time to event information, and 5-year survival status was used as censoring status in survival models. Four survival models were developed and compared in this study: Cox proportional hazards regression model with Elastic Net penalty, random survival forest, gradient boosting survival model and DeepSurv neural network [14].

Survival model performance was assessed using generalisation of area under the receiver operating characteristic (ROC) curve called Harrell’s concordance index (C-index) [23]. The C-index can summarise three different dimensions of predictions (risk, event occurrence and time) in a single number, which provides an easy way to compare performance of different predictive survival models [24]. The interpretation of C-index is identical to the traditional area under the ROC curve (AUC) metric for binary classification: a value of 50% denotes a model that is no better than random and a value of 100% denotes a perfect model. Generally, an AUC statistic of 70–80% is considered good discrimination, 80–90%—excellent discrimination and > 90%—outstanding discrimination [25]. However, it is worth noting that C-index interpretation might be influenced by different level of class imbalance. For example, it can overestimate model performance in severe class imbalance cases or larger amounts of censoring [26]. Two values for C-index were estimated to assess model performance: (1) average C-index from five-fold cross validation using training data; (2) C-index using test data. The best model across different sets of data was chosen based on the C-index calculated on test data.

Four types of survival models were developed for each cancer type separately using four sets of datasets as described in Fig. 1 above. Some algorithms require certain level of data pre-processing such as feature scaling and therefore could not be implemented on all four sets of the data. CoxNet, RSF and GBS models do not require feature scaling, therefore they were trained on all four sets of data. DeepSurv models require feature scaling, therefore they were trained on two sets of data only.

Cox proportional hazards regression model is a widely used semi-parametric regression model that estimates the effect of the features on the survival over time. One of the main advantages of this model is easy interpretable coefficients, however it assumes proportionality of hazards and sometimes is not able to capture complex relationships in high-dimensional data. Penalised Elastic Net Cox proportional hazards model was selected in order to include feature selection in model training process. CoxNet model was implemented using a two-step approach for hyper-parameter tuning. Firstly, CoxNet model was fit on the training data using L1 penalty = 0.8 and default minimum ratio for alpha (0.0001). Secondly, using 100 estimated alphas, hyper-parameter tuning was performed using five-fold cross validation. Grid search was performed to identify best L1 penalty and alpha combination using model performance in terms of C-index.

Random survival forest is an ensemble machine learning method based on decision trees. It is widely used in healthcare research and is known for its flexibility and ability to handle complex relationships between features and survival time in high-dimensional data. Although more complex than CoxNet model, this model provides feature importance that helps improve its interpretability.

RSF model was implemented using a two-step approach for hyper-parameter tuning. Firstly, the model was fit on the training data using default hyper-parameters. Features with permutation importance lower than average were then dropped from the data. Secondly, using reduced feature set, hyper-parameter tuning was performed using five-fold cross validation. Grid search was performed to identify the best combination of the following five parameters using model performance in terms of C-index: maximum depth, maximum features, minimum samples split, minimum samples leaf, maximum samples. Number of trees was set to 100, following guidance in literature stating that the biggest performance gain can often be achieved with the first 100 trees [27]. Larger number of trees was tested for a few models and did not result in better performance. This hyper-parameter grid resulted in 243 candidates fitted over 5 folds, totaling 1215 fits.

A GBS model is similar to RSF, because it relies on multiple base learners to produce overall prediction. It differs from RSF in how the base learners are combined to generate overall prediction. While RSF fits a set of survival trees independently and then averages their predictions, a GBS model is constructed sequentially in a stagewise fashion. Gradient-boosted Cox proportional hazard loss function with regression trees was used as a base learner for GBS model. GBS models were implemented using the same two-step approach for hyper-parameter tuning and setting number of trees to 100 as in RSF models. Grid search was performed to identify the best combination of the following five parameters using model performance in terms of C-index: maximum depth, maximum features, minimum samples split, minimum samples leaf, subsample. This hyper-parameter grid resulted in 243 candidates fitted over 5 folds, totaling 1215 fits.

DeepSurv is a Cox proportional hazards deep neural network introduced by JL Katzman et al. in 2018 [14]. It is a deep feed-forward neural network which predicts the effects of patient’s characteristics on their survival hazard rate parameterised by the weights of the network. It was shown to successfully model complex relationships in prognostic survival modelling and performed as well as or better than other survival models such as linear Cox proportional hazards regression and nonlinear random survival forest. DeepSurv model was implemented using Adam optimiser [28].

DeepSurv model was implemented using a two-step approach for hyper-parameter tuning. Firstly, the model was fit on the training data using default hyper-parameters. Feature importances using SHAP values [29] were then calculated and used for feature selection. Features with feature importance lower than average were then dropped from the data. Secondly, using reduced feature set, hyper-parameter tuning was performed using five-fold cross validation. Optuna study [30] with 100 trials was used for grid search to identify the best combination of the following five parameters using model performance in terms of C-index: number of nodes, dropout rate, learning rate, batch size, number of epochs.

Initial model results with default parameters using DeepSurv were showing significant overfitting, which is expected considering that deep learning models tend to be sensitive to noise and outliers in high-dimensional data. Therefore, two techniques were used to reduce overfitting. Firstly, dropout rate range was set higher than what is commonly used by default. Dropout is a regularisation technique that randomly drops neurons from the model during training. It helps to prevent model overfitting especially for complex models and small datasets. However, setting dropout rate too high might hinder model’s ability to learn from the training data, therefore it is important to find an appropriate level of dropout rate. Secondly, weight decay of 0.001 was set for Adam optimiser to further reduce overfitting. Weight decay adds a penalty to the loss function that discourages large weights, which improves model generalisability and stability. Similarly to dropout rate, setting weight decay too high could lead to underfitting where the model fails to learn important relationships from the training data.

CoxNet, RSF and GBS models were developed using scikit-survival, which is a Python module for survival analysis built on top of scikit-learn module [31]. This module has built-in grid search for hyper-parameter tuning, cross validation and model performance evaluation functions. DeepSurv models were developed using pycox, which is a Python module for survival analysis and time-to-event prediction, built on the torchtuples package for training PyTorch models [32]. Grid search for hyper-parameter tuning and cross validation were implemented using Optuna study [30].

One best model for each cancer type was selected and evaluated using SHAP analysis [29]. Beeswarm plots were created to demonstrate feature importance in the model. These plots include all features in the model, ranked by their importance. The colouring in these plots show the effect direction of the feature (whether higher or lower values are predictive of higher risk of reduced survival). These plots are less useful for visualising the effect of binary features in less complex models such as CoxNet regression, because all SHAP values are distributed at two points (presence of the factor or not). In more complex models such as GBS and RSF, even binary features have broader distribution, because their effect on the prediction depends on the values of other features in the model.

Model predictions were plotted against actuals (actual survival years) in scatter plots. Predicted survival curves were plotted for a few randomly selected patients to demonstrate potential use of the models. Waterfall plots using SHAP values were also plotted for a few randomly selected patients to explain how the model predictions work, based on patient characteristics. To avoid patient identification, feature values were not included in Waterfall plots.

## Results

### Model evaluation

There were 14 different models created for each cancer type, after applying five-fold cross validation and hyper-parameter tuning:

1. CoxNet model on clinical data without feature scaling
2. CoxNet model on clinical data with feature scaling
3. CoxNet model on clinical and genetic data without feature scaling
4. CoxNet model on clinical and genetic data with feature scaling
5. RSF model on clinical data without feature scaling
6. RSF model on clinical data with feature scaling
7. RSF model on clinical and genetic data without feature scaling
8. RSF model on clinical and genetic data with feature scaling
9. GBS model on clinical data without feature scaling
10. GBS model on clinical data with feature scaling
11. GBS survival model on clinical and genetic data without feature scaling
12. GBS model on clinical and genetic data with feature scaling
13. DeepSurv model on clinical data with feature scaling
14. DeepSurv model on clinical and genetic data with feature scaling

**Fig. 2:**
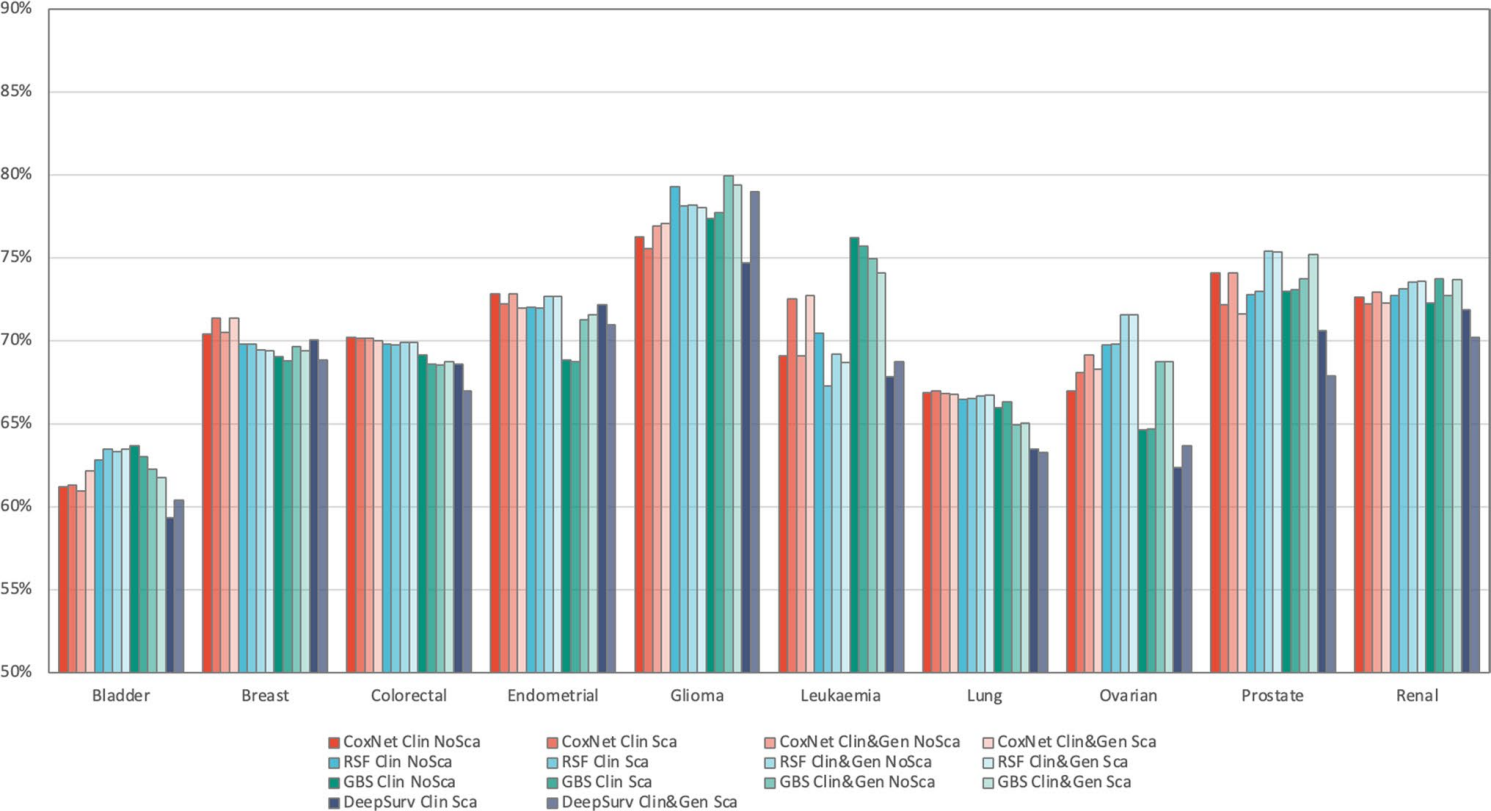
C-index comparison across 14 models by cancer type

Model performance results for four models for each type of machine learning algorithm and each type of dataset are reported in Fig. 2 with an exception of DeppSurv algorithm which only had two models reported. DeepSurv model could only be created with feature scaling applied on the data as described in the Machine learning algorithms section. Model performance results were evaluated by C-index on test data and are shown in Fig. 2. C-index values varied from 60 to 80% with the average C-index across all cancer types of 72%. Most models achieved good performance (C-index > 70%) with an exception of bladder and lung cancers which showed weak predictive ability (best models achieved C-index of 64% and 67% respectively). The best model performance was achieved in glioma with C-index of 80% showing strong predictive ability.

The results on training data, calculated as an average C-index of 5 folds of cross validation, were very similar to the results on test data in all cancer types except for bladder cancer. In bladder cancer, C-index on training data showed better performance compared to test data implying overfitting. C-indices on training data, the results for all 80 models, values of selected hyper-parameters and number of selected features are provided in Supplementary File 2.

Different machine learning algorithms achieved similar performance in most cancer types with CoxNet being the best model in breast, colorectal, endometrial and lung cancers, RSF—in ovarian and prostate cancers, and GBS—in bladder, glioma, leukaemia and renal cancers. In colorectal, endometrial, lung and renal cancers, the model performance difference between CoxNet and RSF methods was marginal. CoxNet model was significantly better than other models in breast cancer. Decision tree-based methods (RSF and GBS) performed significantly better in bladder, glioma and prostate cancers. RSF model was significantly better than other models in ovarian cancer, and GBS model was significantly better than other models in leukaemia. DeepSurv model achieved comparable performance to the other methods, but did not produce the best model in any of the cancer types.

Addition of genetic data improved performance in four cancer types: endometrial, glioma, ovarian and prostate, although the difference in performance was marginal in endometrial cancer. Using genetic data led to the largest increase in model performance in decision tree-based models (RSF and GBS). There were more genetic factors found to be significantly associated with cancer survival in our previous study [17] using Cox model compared to machine learning models in this study. This suggests that the prognostic value of genetic factors is less strong when several powerful clinical factors are included in the model. Genetic features defined as presence of small mutations in single genes might not capture complex genetic interactions well enough to add prognostic value. Also, the frequency of genetic mutations in the patient cohort is generally lower than in clinical factors, which could be another reason why prognostic power of genetic factors is weaker than in clinical factors. Finally, in some cancer types such as renal cancer genetic markers might simply not hold prognostic value.

Feature scaling only marginally improved performance in some models such as breast, lung and renal cancers. This result was not surprising considering that majority of the features included in the models were binary and that machine learning algorithms such as RSF and GBS are able to handle different scale of numeric features.

**Fig. 3:**
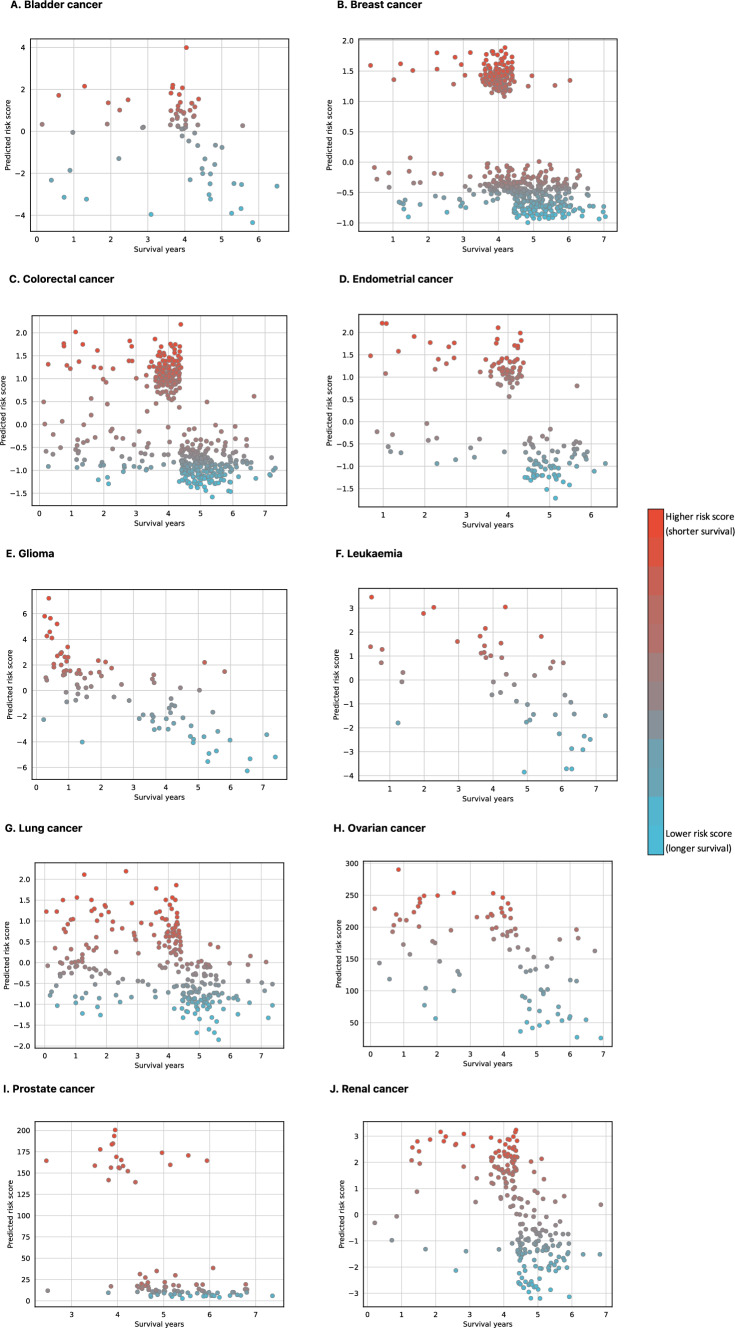
Scatter plots showing relationship between model predicted risk scores and actual survival years

Figure 3 shows model predicted risk scores plotted against actual survival years in patients in the test data, faceted by cancer type. Predicted risk scores are arbitrary values, which do not hold meaning on its own, however, they can be used to rank patients (highest risk score represents shortest survival as predicted by the model).

As seen in Fig. 3, patients with lower predicted scores tend to have longer survival and vice versa showing good model predictive ability across all cancer types. The strongest linear relationship between predicted risk scores and actual survival years in the test data can be seen in glioma and leukaemia models, which is expected considering these models achieved the highest C-indices on test data. Model predictions can also be converted to survival probabilities at different points in time, which allows plotting predicted survival curves (see Fig. 5).

### Prognostic feature importance

The number of features selected in the final model varied from 13 in prostate cancer to 79 in lung cancer with an average number of features of 26. There were two features that were important across all cancer types: referral route and age at diagnosis. Older age was associated with higher risk for shorter survival in all cancers, the effect commonly known from previous studies [33, 34].

**Fig. 4:**
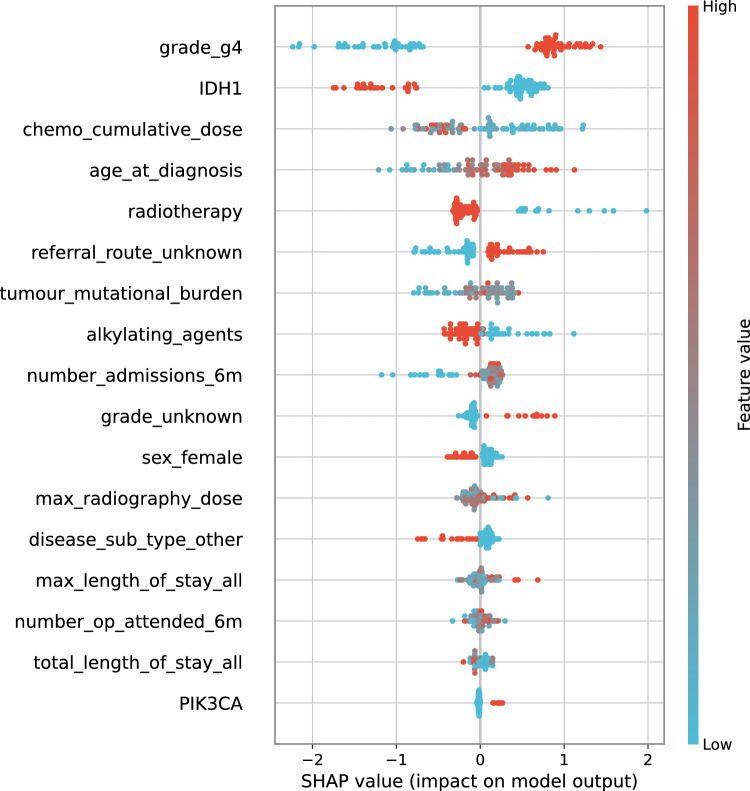
Beeswarm plot summarising feature importance in glioma model

Referral route was the most important feature in all cancer types with the exception of glioma where grade was the most important factor (see Fig. 4 for glioma and Supplementary File 3 for other cancers). Specifically, patients who had unknown referral route were predicted to have shorter survival in all cancer types. This strong effect suggests that missing values in referral route have underlying meaning. Comparing referral route distribution in this data sample with publicly available more recent and better populated national data [35], reveals that missing data in our sample most likely represents patients who had their cancer diagnosed during emergency presentation at hospital or through an urgent GP referral. It is widely known that patients who present acutely have worse survival outcomes [36, 37]. The model results in this study confirmed once again how critical early diagnosis of cancer is for better patient outcomes.

Similarly to referral route, features related to waiting times were important in most cancer types. The results for the effect of waiting times features were mixed across cancer types. Generally, longer waiting times were associated with higher risk for shorter survival, but some cancers showed opposite effect. For example, in prostate cancer longer waiting times were associated with longer survival, which could indicate less advanced cases where treatment is delayed by design.

Stage and grade were important prognostic features in all cancers where staging and grading systems were applicable, which confirmed the usefulness of these systems for cancer prognosis as reported before [5]. Features representing previous hospital utilisation were found to be important for prognosis in all cancer types. In most cases, higher previous hospital utilisation was predictive for worse survival outcomes, but some results were mixed showing that the effect of these features depend on other features in the model.

Multimorbidity was an important prognostic factor in most cancer types, showing association between higher number of pre-existing conditions and shorter survival, which was consistent with the results from previous studies [5]. Individual pre-existing conditions such as anaemia, eye disease, osteoarthritis as well as previous cancer were also found to be good prognostic factors for survival outcomes. Generally, presence of pre-existing conditions was associated with shorter survival.

Radiotherapy was the most important treatment-related feature included in most models. Radiotherapy treatment in bladder, colorectal, endometrial, lung and renal cancers was associated with shorter survival, while in glioma it was associated with longer survival. Over 80% of patients with glioma were treated with radiotherapy, while proportion of patients who had this treatment in other cancers was much lower, for example only 15% in bladder cancer.

Tumour mutational burden (TMB) was an important feature in all four cancers where genetic data added value to model performance. There were mixed results regarding the association of TMB with survival outcomes, suggesting that the effect of TMB on cancer prognosis depends on other features in the model. TP53 gene was the only gene mutation feature that was important in more than one cancer type. Mutations in TP53 gene were associated with worse prognosis in ovarian cancer, while the effect in endometrial cancer was less clear. The effects of mutations in gene TP53 on survival were reported in previous studies [5].

The following features were included in models in at least three cancer types: features related to referral and waiting times (referral route, days from referral to seen, referral to treatment, seen to treatment), demographic features (age at diagnosis, deprivation), tumour-related features (stage, grade, disease sub-type), previous hospital utilisation features (number of outpatient appointments attended, not attended, cancelled, number of hospital admissions [elective, emergency, ordinary and all admissions], number of A&E visits, total and maximum length of stay), features related to pre-existing conditions (multimorbidity, previous cancer, eye disease, anaemia, osteoarthritis, oesophagus disease, pulmonary disease, hernia), treatment-related features (radiotherapy, chemotherapy cumulative dose, alkylating agents), genetic features (TMB, TP53 mutations).

### Model explainability

**Fig. 5:**
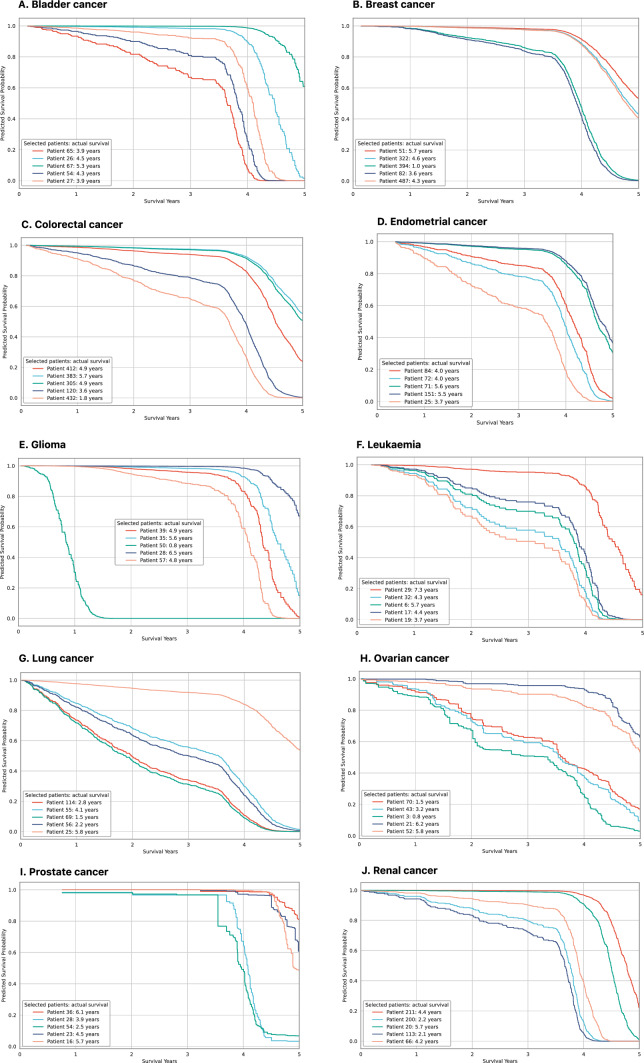
Model predicted survival curves faceted by cancer type

Machine learning models are often seen as “black box” algorithms that are difficult to explain and therefore model predictions are reluctantly used in clinical practice. We provide a few ideas how a prognostic cancer survival model could be used in practice and explain how a prediction is calculated. Figure 5 shows survival curves as predicted by the best machine learning model for each cancer type for five randomly selected patients. The legend on the graph shows the actual survival years for each patient. As seen in the figure, generally models predicted longer survival for those patients who actually survived longer. For example, in colorectal cancer (Part C in Fig. 5), Patient 383 was predicted to have more than 80% probability of surviving 4 years and more than 50% probability of surviving 5 years, while actual survival was 5.7 years. In comparison, Patient 432 was predicted to have just over 60% probability of surviving 3 years and about 30% probability of surviving 4 years, while actual survival was 1.8 years.

**Fig. 6:**
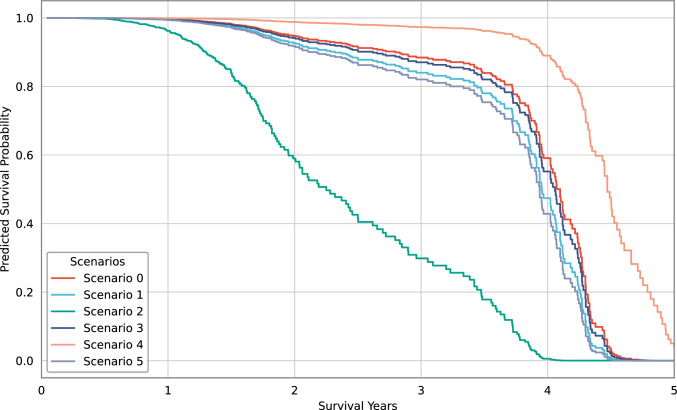
Predicted survival curves in hypothetical scenarios using glioma survival model

Hypothetical survival curves could be created to demonstrate how model predicted survival probabilities would change if patient’s characteristics were different. For example, Fig. 6 shows predicted survival curves for one patient with glioma cancer in different scenarios. Scenario 0 represents actual information about the patient: young patient in their 20s with low grade glioma with unknown referral method, no mutations in gene IDH1 and no treatment with alkylating agents. The actual survival for this patient was 4.8 years. The following hypothetical scenarios were applied to demonstrate changes in predicted survival curves:

1. Scenario 1 – patient is older (60 years old)
2. Scenario 2 – patient has high grade glioma (grade 4)
3. Scenario 3 – patient is treated with alkylating agents
4. Scenario 4 – patient was referred through GP
5. Scenario 5 – patient has a mutation in gene IDH1

As shown in Fig. 6, older age, higher grade, treatment with alkylating agents and mutation in gene IDH1 reduced predicted survival, while referral method through GP increased predicted survival. This type of visualisation allows to clearly demonstrate the effect of different factors on overall survival. For example, it can be seen that if glioma was diagnosed late and was higher grade, the survival probabilities would be significantly lower.

These predicted survival curves could be used in clinical practice to inform about patient’s prognosis. Using predicted probabilities at different points in time is preferable over having a specific prediction of number of years (or months), because it provides more information and makes it clearer that prediction includes some uncertainty.

**Fig. 7:**
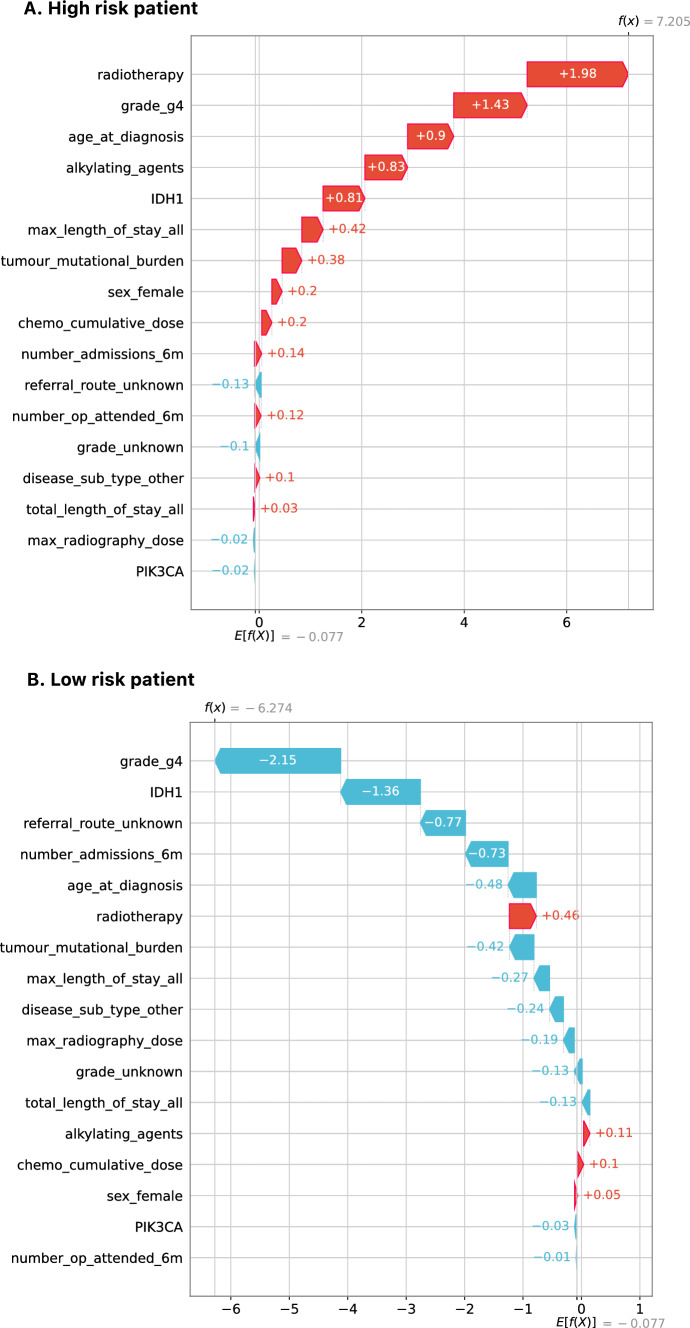
Waterfall plot for high risk and low risk patients as predicted by glioma model

Each model prediction can be explained using a Waterfall plot (see Fig. 7 for glioma and Supplementary File 4 for other cancers). The figure shows contribution of all model features towards the model prediction for one high risk and one low risk patient in glioma. To avoid patient identification, actual feature values were removed from the plot, making it difficult to interpret the effect direction (whether risk is increased because patient has or does not have that feature). Therefore, these plots were produced only for demonstration purposes.

It can be seen from Part A in Fig. 7 that radiotherapy, grade and age are the biggest contributors to high risk for this patient. It can also be seen from the graph that other contributors to the predicted high risk are treatment with alkylating agents, mutations in IDH1 gene, maximum length of stay and TMB features. Waterfall charts could be used in clinical practice to explain model predictions on individual patient level, which might help convince health practitioners about model’s validity.

## Discussion

The amount of healthcare data being collected and made available to researchers is increasing exponentially, which has led to the rise of application of machine learning and similar computational methods in healthcare. However, the systematic review we conducted previously [5] showed that most of the research completed on prognostic factors related to cancer survival were based on traditional regression methods, such as Cox proportional hazards regression. The potential of using more advanced computational methods such as machine learning remains unexplored in this area.

Traditional statistical models such as Cox proportional hazards regression are usually aimed at inferring relationships between the factors and the outcome. Machine learning methods are usually focused at making accurate predictions about the outcome when taking into account the predictive factors. Machine learning methods are also more flexible when dealing with imperfect datasets and more scalable when dealing with large amounts of data and a high level of dimensionality. This makes machine learning methods more appropriate for deployment and use in clinical practice compared to conventional statistical approaches [38]. On the contrary, more traditional regression methods often offer better interpretability of the model results and predictions, which is also an important factor for application and adoption of the predictive models in clinical practice [39, 40]. Therefore, different types of predictive algorithms were applied and compared in this study. Namely, the results from four predictive machine learning models, including regression based CoxNet model, tree-based Random Forest Survival and Gradient Boosting Survival models, and deep learning DeepSurv neural network model were evaluated and compared on the data of ten cancer types. To address common criticism for low interpretability of machine learning models, we provided examples of how these models can be interpreted and used in clinical practice.

From methodological perspective, this study provides a comparison of different missing data imputation methods and assesses the impact of feature scaling. Multivariate feature imputation with decision tree regressor was found to be the most accurate method for missing data imputation in this dataset. Feature scaling improved performance in three out of ten cancer types, although improvement was marginal, suggesting that machine learning algorithms such as random survival forest or gradient boosting survival are able to handle features at different scales. In addition, the interpretation of model results is more difficult when features are scaled, therefore the minor benefits of feature scaling might not outweigh the disadvantages.

There were 140 predictive machine learning survival models created in total after applying five-fold cross validation and hyper-parameter tuning (14 models for each cancer type). Most models achieved good predictive ability with C-index above 70%. The best model performance was achieved in glioma, leukaemia and prostate cancers, while the worst model performance was in bladder and lung cancers. Generally, different machine learning methods achieved comparable model performance with tree-based models (RSF and GBS) performing better across most cancer types (bladder, glioma, leukaemia, ovarian and prostate cancers). CoxNet model was significantly better than other models in breast cancer. DeepSurv neural networks produced similar or worse performance to other machine learning methods. The important features selected in predictive models were similar across different machine learning methods within the same cancer type and comparable across different cancer types.

Referral route was one of the most important predictive features in all models across different cancer types, implying that emergency presentation is predictive of shorter survival. Features representing waiting times such as days from referral to treatment were also found to be important prognostic features in most models. Similarly, the findings demonstrated the predictive power of features related to previous hospital utilisation, in most cases showing relation of higher hospital utilisation with shorter survival. These effects on cancer survival were not widely explored before and could be useful when determining cancer prognosis [5].

Potential biases and inequalities in access to healthcare should be considered when analysing the impact of referral routes and waiting times on cancer survival. Although the patient cohort is based on patients living in the UK where the NHS prioritises equal access to healthcare for all, it is known that disparities do exist in certain demographic groups. Some patients might be disadvantaged when accessing healthcare services depending on their socioeconomic status, geographic location or ethnic background. There could be confounding factors related to patient demographics that are masked by emergency presentation and late diagnosis, which could be affecting overall survival. Machine learning models developed in this study included demographic factors such as ethnicity and deprivation, but more detailed information representing socioeconomic status, disabilities and other demographic characteristics could be considered in the future to further reduce potential biases. Other statistical approaches such as propensity score matching [41] could also be applied to minimize the effect of demographics when constructing the models.

Age, stage, grade and disease sub-type were important predictive features in most machine learning models, which was consistent with the results from previous literature [5]. Similarly, multimorbidity was found to be an important prognostic factor in most cancer types. The findings also identified the prognostic importance of some pre-existing conditions such as anaemia, eye disease, osteoarthritis, pulmonary disease, hernia, hypertension and previous cancer. Treatment-related features such as radiotherapy, chemotherapy cumulative dose and alkylating agents were also important in predictive machine learning models, but the effect direction depended on cancer type and interaction with other factors. This shows that once cancer treatment is determined, it might help estimate prognosis more accurately.

Genetic features were included in predictive models only in four cancer types: endometrial, glioma, ovarian and prostate. Tumour mutational burden feature was important in all four cancers confirming its strong predictive power as shown in previous studies [5]. Mutations in TP53 gene were predictive of shorter survival in endometrial and ovarian cancers, which was consistent with the results reported previously [42, 43]. Mutations in IDH1 gene were predictive of longer survival in glioma, which was also reported previously [44]. There were two genetic features found to be predictive of shorter survival, namely FBXW7 in endometrial cancer and PIK3CA in glioma, which were not reported before [5]. It is known that FBXW7 gene is frequently mutated in endometrial cancer [45] and machine learning models demonstrated its importance for predicting endometrial cancer prognosis. PIK3CA is one of the most common mutated genes in patients with cancer and its association with cancer survival was investigated before with results showing no impact on survival [46]. There were fewer genetic factors with strong prognostic power found in machine learning models compared to simpler Cox proportional hazards regression model reported before [17], suggesting that single gene features might not capture complex genetic relationships as well as some strong clinical factors. In some cancer types such as renal cancer, genetic markers might simply not have strong prognostic value and therefore clinicopathological factors should be the focus when modelling cancer survival.

### Limitations

Several limitations were recognised in the data used in this project. More advanced metastatic cancers were excluded from the analysis and the majority of genetic samples collected from the participants of the 100,000 Genomes Project were taken from surgical resection showing that most patients in the cohort had surgical treatment. This patient cohort is therefore representative of generally less advanced cancer cases. Although most of the important prognostic factors such as age, stage, grade, referral route, multimorbidity would likely have similar prognostic power in more advanced cancers, some factors might be different. Namely, treatment-related factors such as chemotherapy or radiotherapy dose would likely have different effect on survival outcomes [47]. Also, certain prognostic factors found to be strong predictors in this patient cohort could be even more important in more advanced cancers. For example, waiting times are likely to have even stronger impact on survival outcomes in higher stage and metastatic cancers.

Another limitation of the data is small sample sizes in certain cancer types. There were more patients in the cohort with more common cancer types such as breast, colorectal and lung cancer, while fewer patients for less common cancer types such as leukaemia, glioma, bladder and ovarian cancer. Machine learning models are known to work better when the size of training data is large, therefore having larger datasets for less common cancer types could improve model performance and help identify more prognostic associations with overall survival. Having larger datasets might also help discover more significant relationships between genetic biomarkers and cancer survival, because genetic mutation frequencies tend to be low and require larger sample size to draw reliable conclusions.

Although several data sources were linked to obtain a comprehensive cancer dataset in this study, other data sources such as General Practice (GP) data could add value in predictive survival modelling. For example, long-term conditions and certain behavioural factors such as smoking, alcohol use, BMI are better captured in GP data. In addition, this study focused on SNVs and small insertions and deletions (indels) for assessing the prognostic power of genetic mutations for cancer survival. In the future, other types of genetic mutations could be tested such as copy number variations or structural variations.

Another limitation of the data used in this study was high level of data missingness observed for some variables such as tumour size, referral route, waiting times, chemotherapy dose and radiography dose. Analyses conducted in this study were predominantly based on categorical variables, therefore missing data were assigned a separate ‘Unknown’ category. Unknown category for referral route was identified as the most important prognostic factor in most machine learning survival models suggesting that the missingness in this variable has an underlying prognostic meaning, and most likely masks emergency referral routes. Different missing data imputation techniques were tested by comparing data distributions using visualization techniques and applied for numeric features to reduce the impact of missing data on the results. Further analysis of different missing data imputation techniques could be conducted in the future using statistical tests for differences in data distributions.

Machine learning survival model performance was evaluated using C-index, which has become a gold standard for measuring performance of such models and has been highly influential for scrutinising predictive models [48]. However, there are a few known limitations of C-index. The index value depends on the ranks of the predicted values, therefore models with consistently inaccurate predictions might have a larger index value than a model with more accurate predictions overall, but less accurate ranking. If predictive survival models are used in practice to predict survival years as opposed to distinguish between lower and higher risk patients, it would be advisable to estimate model calibration metrics to ensure the model predictions are accurate. C-index is an overall metric that combines model performance information such as sensitivity and specificity, but the separate concepts of sensitivity and specificity could be meaningful in clinical practice.

Machine learning models learn the important relationships from the data which changes over time, especially in rapidly evolving fields like oncology. Therefore, it is extremely important to continuously monitor and update (re-train) the prognostic survival models to maintain their accuracy and predictive power. These models could be updated by adding other data sources, including new prognostic factors and using alternative algorithms as new clinical and genetic insights and treatment methods emerge. When used in clinical practice, monitoring model performance over time could help decide when the model needs to be updated with more recent data. Machine learning models developed in this study could also be validated on external datasets to demonstrate their predictive power and generalizability.

## Conclusions

Several machine learning survival models for each cancer type were developed in this study showing good or even excellent discrimination [25]. These prognostic models could be used in clinical practice to help determine cancer prognosis more accurately compared to using staging and grading systems alone. These models could be used at the time of cancer diagnosis or once the treatment plan is confirmed. Predicted survival curves provide a good way of showing prognosis as survival probabilities at points in time rather than providing a specific number of years.

Despite the potential of artificial intelligence to improve healthcare services and patient outcomes, adoption in clinical practice has been slow and there remain a few barriers [49]. Technology is one of the main barriers for the machine learning model adoption in the NHS with challenges such as limited data quality, data access and interoperability. The UK government recently published guidance outlining their expectations for artificial intelligence applied in the NHS, which might support its adoption in the future [50]. The guidance emphasised the need for transparency and explainability of machine learning algorithms. Clinician “buy-in” and engagement remains one of the main barriers for artificial intelligence adoption in clinical practice. Clinicians often have limited confidence and trust in complex predictive models, which are often considered as “black box” algorithms without clear explanation on how model makes its decisions. Therefore, improving model interpretability is key for increasing trust and adoption of such models. Model explainability methods such as feature importances and waterfall charts based on SHAP values as well as predicted survival curves using hypothetical scenarios help interpret the model prediction. Alternatively, results and predictions from complex machine learning models can be compared to simpler models such as traditional regression models that are more widely understood by medical staff.

In summary, the results from this study contribute to the understanding of cancer disease and could be used by researchers to further test and build the knowledge base about prognostic factors for cancer survival. The findings draw attention to certain healthcare practice-related prognostic factors such as referral route, waiting times and previous hospital encounters emphasising the importance of early cancer diagnosis and timely treatment. Predictive machine learning models could be used in clinical practice to increase the accuracy of cancer prognosis and consequently contribute to the improvement of patient outcomes.

## Supplementary Information

Additional file 1

## Data Availability

The data that support the findings of this study are available in Genomics England’s cloud-based workspace called Research Environment, but restrictions apply to the access of these data. Python code used to develop prognostic models are available from the corresponding author on reasonable request.
