# Supplementary material for: Pan-cancer predictive survival model development and evaluation using electronic health record and genetic data across 10 cancer types
Source: Discov Oncol. 2025 May 12;16:735. doi: 10.1007/s12672-025-02523-1 (PMC12069196; doi:10.1007/s12672-025-02523-1)
Supplement: Supplementary file 1 — Additional file 1 [file 12672_2025_2523_MOESM1_ESM.docx]

**Supplementary File 1.** List of features considered in predictive models

| **Feature** | **Category** | **Type** | **Data Source** | **Bladder** | **Breast** | **Colorectal** | **Endometrial** | **Glioma** | **Leukaemia** | **Lung** | **Ovarian** | **Prostate** | **Renal** | **No Cancers** |
| --- | --- | --- | --- | --- | --- | --- | --- | --- | --- | --- | --- | --- | --- | --- |
| **Age** |  | Numeric | Genomics England | x | x | x | x | x | x | x | x | x | x | 10 |
| **Sex female** |  | Binary | Genomics England | x |  | x |  | x | x | x |  |  | x | 6 |
| **Ethnicity** | Asian | Categorical | Genomics England, NCRAS, HES |  | x | x | x | x |  | x | x | x | x | 8 |
| **Ethnicity** | Black | Categorical | Genomics England, NCRAS, HES |  | x | x | x |  |  | x |  | x | x | 6 |
| **Ethnicity** | White | Categorical | Genomics England, NCRAS, HES |  | ref | ref | ref | ref |  | ref | ref | ref | ref | 8 |
| **Ethnicity** | Mixed | Categorical | Genomics England, NCRAS, HES |  | x |  |  |  |  |  |  |  |  | 1 |
| **Ethnicity** | Other | Categorical | Genomics England, NCRAS, HES |  | x | x | x | x |  | x | x |  | x | 7 |
| **Ethnicity** | Unknown | Categorical | Genomics England, NCRAS, HES |  | x | x | x |  |  | x | x | x | x | 7 |
| **Deprivation** | 1 Least Deprived | Categorical | NCRAS, HES | ref | ref | ref | ref | ref | ref | ref | ref | ref | ref | 10 |
| **Deprivation** | 2 Second Least Deprived | Categorical | NCRAS, HES | x | x | x | x | x | x | x | x | x | x | 10 |
| **Deprivation** | 3 Third Most Deprived | Categorical | NCRAS, HES | x | x | x | x | x | x | x | x | x | x | 10 |
| **Deprivation** | 4 Second Most Deprived | Categorical | NCRAS, HES | x | x | x | x | x | x | x | x | x | x | 10 |
| **Deprivation** | 5 Most Deprived | Categorical | NCRAS, HES | x | x | x | x | x | x | x | x | x | x | 10 |
| **Deprivation** | Unknown | Categorical | NCRAS, HES |  | x |  |  |  |  |  |  |  |  | 1 |
| **Disease subtype** | Acute lymphoblastic leukaemia | Categorical | Genomics England |  |  |  |  |  | x |  |  |  |  | 1 |
| **Disease subtype** | Acute myeloid leukaemia | Categorical | Genomics England |  |  |  |  |  | ref |  |  |  |  | 1 |
| **Disease subtype** | Adenocarcinoma | Categorical | Genomics England |  |  |  |  |  |  | ref |  |  |  | 1 |
| **Disease subtype** | Astrocytoma | Categorical | Genomics England |  |  |  |  | x |  |  |  |  |  | 1 |
| **Disease subtype** | Chromophobe renal cell | Categorical | Genomics England |  |  |  |  |  |  |  |  |  | x | 1 |
| **Disease subtype** | Chronic lymphocytic leukaemia | Categorical | Genomics England |  |  |  |  |  | x |  |  |  |  | 1 |
| **Disease subtype** | Clear cell | Categorical | Genomics England |  |  |  |  |  |  |  |  |  | ref | 1 |
| **Disease subtype** | Ductal | Categorical | Genomics England |  | ref |  |  |  |  |  |  |  |  | 1 |
| **Disease subtype** | Endometrioid adenocarcinoma | Categorical | Genomics England |  |  |  | ref |  |  |  | x |  |  | 2 |
| **Disease subtype** | Glioblastoma | Categorical | Genomics England |  |  |  |  | ref |  |  |  |  |  | 1 |
| **Disease subtype** | Lobular | Categorical | Genomics England |  | x |  |  |  |  |  |  |  |  | 1 |
| **Disease subtype** | Papillary | Categorical | Genomics England |  |  |  |  |  |  |  |  |  | x | 1 |
| **Disease subtype** | Serous carcinoma | Categorical | Genomics England |  |  |  | x |  |  |  | ref |  |  | 2 |
| **Disease subtype** | Squamous cell | Categorical | Genomics England |  |  |  |  |  |  | x |  |  |  | 1 |
| **Disease subtype** | Other | Categorical | Genomics England |  | x |  | x | x | x | x | x |  | x | 7 |
| **Tumour site** | Ascending colon | Categorical | NCRAS |  |  | x |  |  |  |  |  |  |  | 1 |
| **Tumour site** | Caecum | Categorical | NCRAS |  |  | x |  |  |  |  |  |  |  | 1 |
| **Tumour site** | Central portion | Categorical | NCRAS |  | x |  |  |  |  |  |  |  |  | 1 |
| **Tumour site** | Descending colon | Categorical | NCRAS |  |  | x |  |  |  |  |  |  |  | 1 |
| **Tumour site** | Frontal lobe | Categorical | NCRAS |  |  |  |  | ref |  |  |  |  |  | 1 |
| **Tumour site** | Hepatic flexure of colon | Categorical | NCRAS |  |  | x |  |  |  |  |  |  |  | 1 |
| **Tumour site** | Lower lobe | Categorical | NCRAS |  |  |  |  |  |  | x |  |  |  | 1 |
| **Tumour site** | Lower-inner quadrant | Categorical | NCRAS |  | x |  |  |  |  |  |  |  |  | 1 |
| **Tumour site** | Lower-outer quadrant | Categorical | NCRAS |  | x |  |  |  |  |  |  |  |  | 1 |
| **Tumour site** | Multiple sites | Categorical | NCRAS |  | x | x |  |  |  |  |  |  |  | 2 |
| **Tumour site** | Overlapping lesion | Categorical | NCRAS |  | x |  |  |  |  |  |  |  |  | 1 |
| **Tumour site** | Parietal lobe | Categorical | NCRAS |  |  |  |  | x |  |  |  |  |  | 1 |
| **Tumour site** | Rectosigmoid junction | Categorical | NCRAS |  |  | x |  |  |  |  |  |  |  | 1 |
| **Tumour site** | Rectum | Categorical | NCRAS |  |  | ref |  |  |  |  |  |  |  | 1 |
| **Tumour site** | Sigmoid colon | Categorical | NCRAS |  |  | x |  |  |  |  |  |  |  | 1 |
| **Tumour site** | Temporal lobe | Categorical | NCRAS |  |  |  |  | x |  |  |  |  |  | 1 |
| **Tumour site** | Transverse colon | Categorical | NCRAS |  |  | x |  |  |  |  |  |  |  | 1 |
| **Tumour site** | Upper lobe | Categorical | NCRAS |  |  |  |  |  |  | ref |  |  |  | 1 |
| **Tumour site** | Upper-inner quadrant | Categorical | NCRAS |  | x |  |  |  |  |  |  |  |  | 1 |
| **Tumour site** | Upper-outer quadrant | Categorical | NCRAS |  | ref |  |  |  |  |  |  |  |  | 1 |
| **Tumour site** | Other | Categorical | NCRAS |  | x | x |  | x |  | x |  |  |  | 4 |
| **Tumour size** |  | Numeric | NCRAS |  | x |  |  |  |  | x |  |  | x | 3 |
| **Previous cancer** |  | Binary | NCRAS | x | x | x | x | x | x | x | x | x | x | 10 |
| **Grade** | G1 | Categorical | NCRAS | ref | ref | ref | ref |  |  | ref | ref |  | ref | 7 |
| **Grade** | G2 | Categorical | NCRAS | x | x | x | x | ref |  | x | x | ref | x | 9 |
| **Grade** | G3 | Categorical | NCRAS | x | x | x | x | x |  | x | x | x | x | 9 |
| **Grade** | G4 | Categorical | NCRAS |  |  |  |  | x |  |  |  |  | x | 2 |
| **Grade** | Unknown | Categorical | NCRAS | x | x | x | x | x |  | x | x | x | x | 9 |
| **TNM Stage** | Stage 0 | Categorical | NCRAS | ref |  |  |  |  |  |  |  |  |  | 1 |
| **TNM Stage** | Stage 1 | Categorical | NCRAS | x |  |  | ref |  |  |  | ref | ref | ref | 5 |
| **TNM Stage** | Stage 2 | Categorical | NCRAS | x |  |  | x |  |  |  | x | x | x | 5 |
| **TNM Stage** | Stage 3 | Categorical | NCRAS | x |  |  | x |  |  |  | x | x | x | 5 |
| **TNM Stage** | Unknown | Categorical | NCRAS |  |  |  | x |  |  |  |  | x | x | 3 |
| **T stage** | Stage 0 | Categorical | NCRAS |  |  |  |  |  |  |  |  |  |  | 0 |
| **T stage** | Stage 1 | Categorical | NCRAS |  | ref | ref |  |  |  | ref |  |  |  | 3 |
| **T stage** | Stage 2 | Categorical | NCRAS |  | x | x |  |  |  | x |  |  |  | 3 |
| **T stage** | Stage 3 | Categorical | NCRAS |  | x | x |  |  |  | x |  |  |  | 3 |
| **T stage** | Stage 4 | Categorical | NCRAS |  | x | x |  |  |  | x |  |  |  | 3 |
| **T stage** | Unknown | Categorical | NCRAS |  | x | x |  |  |  | x |  |  |  | 3 |
| **N stage** | Stage 0 | Categorical | NCRAS |  | ref | ref |  |  |  | ref |  |  |  | 3 |
| **N stage** | Stage 1 | Categorical | NCRAS |  | x | x |  |  |  | x |  |  |  | 3 |
| **N stage** | Stage 2 | Categorical | NCRAS |  | x | x |  |  |  | x |  |  |  | 3 |
| **N stage** | Stage 3 | Categorical | NCRAS |  | x |  |  |  |  | x |  |  |  | 2 |
| **N stage** | Unknown | Categorical | NCRAS |  | x | x |  |  |  | x |  |  |  | 3 |
| **NPI score** |  | Numeric | NCRAS |  | x |  |  |  |  |  |  |  |  | 1 |
| **Dukes stage** | A | Categorical | NCRAS |  |  | ref |  |  |  |  |  |  |  | 1 |
| **Dukes stage** | B | Categorical | NCRAS |  |  | x |  |  |  |  |  |  |  | 1 |
| **Dukes stage** | C | Categorical | NCRAS |  |  | x |  |  |  |  |  |  |  | 1 |
| **Dukes stage** | D | Categorical | NCRAS |  |  |  |  |  |  |  |  |  |  | 0 |
| **Dukes stage** | Unknown | Categorical | NCRAS |  |  | x |  |  |  |  |  |  |  | 1 |
| **Figo stage** | Stage 1 | Categorical | NCRAS |  |  |  | ref |  |  |  | ref |  |  | 2 |
| **Figo stage** | Stage 2 | Categorical | NCRAS |  |  |  | x |  |  |  | x |  |  | 2 |
| **Figo stage** | Stage 3 | Categorical | NCRAS |  |  |  | x |  |  |  | x |  |  | 2 |
| **Figo stage** | Stage 4 | Categorical | NCRAS |  |  |  |  |  |  |  |  |  |  | 0 |
| **Figo stage** | Unknown | Categorical | NCRAS |  |  |  | x |  |  |  | x |  |  | 2 |
| **Gleason score** |  | Numeric | NCRAS |  |  |  |  |  |  |  |  | x |  | 1 |
| **Nodes involved** |  | Numeric | NCRAS |  | x | x |  |  |  | x |  |  |  | 3 |
| **Laterality** | Bilateral | Categorical | NCRAS |  | x |  |  |  |  |  | ref |  |  | 2 |
| **Laterality** | Left | Categorical | NCRAS |  | ref |  |  | x |  | x | x |  | ref | 5 |
| **Laterality** | Midline | Categorical | NCRAS |  |  |  |  |  |  |  |  |  |  | 0 |
| **Laterality** | Right | Categorical | NCRAS |  | x |  |  | ref |  | ref | x |  | x | 5 |
| **Laterality** | Unknown | Categorical | NCRAS |  | x |  |  | x |  | x | x |  | x | 5 |
| **ER status** | Positive | Categorical | NCRAS |  | ref |  |  |  |  |  |  |  |  | 1 |
| **ER status** | Negative | Categorical | NCRAS |  | x |  |  |  |  |  |  |  |  | 1 |
| **ER status** | Unknown | Categorical | NCRAS |  | x |  |  |  |  |  |  |  |  | 1 |
| **PR status** | Positive | Categorical | NCRAS |  | ref |  |  |  |  |  |  |  |  | 1 |
| **PR status** | Negative | Categorical | NCRAS |  | x |  |  |  |  |  |  |  |  | 1 |
| **PR status** | Unknown | Categorical | NCRAS |  | x |  |  |  |  |  |  |  |  | 1 |
| **HER2 status** | Positive | Categorical | NCRAS |  | x |  |  |  |  |  |  |  |  | 1 |
| **HER2 status** | Negative | Categorical | NCRAS |  | ref |  |  |  |  |  |  |  |  | 1 |
| **HER2 status** | Unknown | Categorical | NCRAS |  | x |  |  |  |  |  |  |  |  | 1 |
| **Multifocal** |  | Binary | NCRAS |  | x |  |  |  |  |  |  |  |  | 1 |
| **Referral route** | Emergency presentation | Categorical | NCRAS | x | x | x | x | x | x | x | x |  | x | 9 |
| **Referral route** | GP referral | Categorical | NCRAS | ref | x | x | x | x | ref | x | x | x | x | 10 |
| **Referral route** | Inpatient elective | Categorical | NCRAS |  |  | x |  |  |  |  |  |  | x | 2 |
| **Referral route** | Other outpatient | Categorical | NCRAS | x | x | x | x | ref | x | x | x | x | x | 10 |
| **Referral route** | Screening | Categorical | NCRAS |  | x | x |  |  |  |  |  |  |  | 2 |
| **Referral route** | TWW | Categorical | NCRAS | x | ref | ref | ref |  | x | ref | ref | ref | ref | 9 |
| **Referral route** | Unknown | Categorical | NCRAS | x | x | x | x | x | x | x | x | x | x | 10 |
| **Referral to seen** |  | Numeric | NCRAS | x | x | x | x |  |  | x | x | x | x | 8 |
| **Seen to treatment** |  | Numeric | NCRAS | x | x | x | x | x | x | x | x | x | x | 10 |
| **Referral to treatment** |  | Numeric | NCRAS | x | x | x | x |  |  | x | x | x | x | 8 |
| **Tumour mutational burden** |  | Numeric | Genomics England | x | x | x | x | x | x | x | x | x | x | 10 |
| **Alkylating agents** |  | Binary | NCRAS | x | x | x | x | x | x | x | x |  | x | 9 |
| **Antimetabolites** |  | Binary | NCRAS | x | x | x |  |  | x | x | x |  |  | 6 |
| **Antitumour agents** |  | Binary | NCRAS |  | x |  | x |  | x |  | x |  |  | 4 |
| **Hormone therapy** |  | Binary | NCRAS |  | x |  |  |  |  |  |  | x |  | 2 |
| **Kinase inhibitors** |  | Binary | NCRAS |  | x |  |  |  | x | x |  |  | x | 4 |
| **Plant alkaloids** |  | Binary | NCRAS |  | x | x | x | x |  | x | x |  |  | 6 |
| **Targeted and immunotherapy** |  | Binary | NCRAS | x | x | x |  |  | x | x | x |  | x | 7 |
| **Other chemotherapy** |  | Binary | NCRAS | x | x |  |  |  | x | x |  |  | x | 5 |
| **Radiotherapy** |  | Binary | NCRAS | x | x | x | x | x | x | x | x | x | x | 10 |
| **Chemo cumulative dose** |  | Numeric | NCRAS |  | x |  |  | x | x |  | x |  |  | 4 |
| **Max teletherapy fields** |  | Numeric | NCRAS |  | x |  | x | x |  |  |  |  |  | 3 |
| **Max radiography dose** |  | Numeric | NCRAS |  | x |  | x | x |  |  |  |  |  | 3 |
| **Number of admissions (all)** |  | Numeric | HES |  | x | x |  |  |  |  |  |  | x | 3 |
| **Number of ordinary admissions (all)** |  | Numeric | HES |  | x | x |  |  |  | x | x | x | x | 6 |
| **Number of elective admissions (all)** |  | Numeric | HES | x | x | x | x | x |  | x | x |  | x | 8 |
| **Number of emergency admissions (all)** |  | Numeric | HES |  | x | x |  |  | x |  |  |  | x | 4 |
| **Max length of stay (all)** |  | Numeric | HES | x |  |  |  | x |  | x | x | x |  | 5 |
| **Total length of stay (all)** |  | Numeric | HES |  |  |  |  | x |  | x |  | x |  | 3 |
| **Number of A&E visits (all)** |  | Numeric | HES |  | x | x | x |  |  | x |  | x | x | 6 |
| **Number of A&E visits by ambulance (all)** |  | Numeric | HES |  | x | x | x | x |  | x | x |  | x | 7 |
| **Number of attended outpatient appts (all)** |  | Numeric | HES | x | x | x | x |  | x | x |  | x | x | 8 |
| **Number of DNA & cancelled outpatient appts (all)** |  | Numeric | HES | x | x | x | x |  |  | x |  |  |  | 5 |
| **Number of admissions (5y)** |  | Numeric | HES | x |  |  |  |  | x |  |  |  |  | 2 |
| **Number of ordinary admissions (5y)** |  | Numeric | HES | x |  |  |  |  |  |  |  |  |  | 1 |
| **Number of elective admissions (5y)** |  | Numeric | HES |  |  |  |  |  | x |  |  |  |  | 1 |
| **Number of emergency admissions (5y)** |  | Numeric | HES |  |  |  |  | x |  |  |  |  |  | 1 |
| **Max length of stay (5y)** |  | Numeric | HES |  | x | x |  |  |  |  |  |  |  | 2 |
| **Total length of stay (5y)** |  | Numeric | HES |  | x |  |  |  | x |  |  |  |  | 2 |
| **Number of A&E visits (5y)** |  | Numeric | HES |  |  |  |  |  |  |  |  |  |  | 0 |
| **Number of A&E visits by ambulance (5y)** |  | Numeric | HES |  |  |  |  |  |  |  |  |  |  | 0 |
| **Number of attended outpatient appts (5y)** |  | Numeric | HES |  |  |  |  |  |  |  |  |  |  | 0 |
| **Number of DNA & cancelled outpatient appts (5y)** |  | Numeric | HES |  |  |  |  |  | x |  |  | x |  | 2 |
| **Number of admissions (1y)** |  | Numeric | HES |  |  |  | x |  |  |  | x |  |  | 2 |
| **Number of ordinary admissions (1y)** |  | Numeric | HES |  |  |  | x |  | x |  |  |  |  | 2 |
| **Number of elective admissions (1y)** |  | Numeric | HES |  |  |  |  |  |  |  |  |  |  | 0 |
| **Number of emergency admissions (1y)** |  | Numeric | HES | x |  |  | x |  |  | x |  |  |  | 3 |
| **Max length of stay (1y)** |  | Numeric | HES |  |  |  | x |  |  |  |  |  | x | 2 |
| **Total length of stay (1y)** |  | Numeric | HES |  |  |  |  |  |  |  |  |  | x | 1 |
| **Number of A&E visits (1y)** |  | Numeric | HES |  |  |  |  |  | x |  |  |  |  | 1 |
| **Number of A&E visits by ambulance (1y)** |  | Numeric | HES | x |  |  |  |  |  |  |  | x |  | 2 |
| **Number of attended outpatient appts (1y)** |  | Numeric | HES |  |  |  |  |  |  |  |  |  |  | 0 |
| **Number of DNA & cancelled outpatient appts (1y)** |  | Numeric | HES |  |  |  |  |  |  |  |  |  |  | 0 |
| **Number of admissions (6m)** |  | Numeric | HES |  |  |  |  | x |  | x |  | x |  | 3 |
| **Number of ordinary admissions (6m)** |  | Numeric | HES |  |  |  |  | x |  |  |  |  |  | 1 |
| **Number of elective admissions (6m)** |  | Numeric | HES |  |  |  |  |  |  |  |  | x |  | 1 |
| **Number of emergency admissions (6m)** |  | Numeric | HES |  |  |  |  |  |  |  | x | x |  | 2 |
| **Max length of stay (6m)** |  | Numeric | HES |  |  |  |  |  | x |  |  |  |  | 1 |
| **Total length of stay (6m)** |  | Numeric | HES | x |  | x | x |  |  |  | x |  |  | 4 |
| **Number of A&E visits (6m)** |  | Numeric | HES | x |  |  |  | x |  |  | x |  |  | 3 |
| **Number of A&E visits by ambulance (6m)** |  | Numeric | HES |  |  |  |  |  | x |  |  |  |  | 1 |
| **Number of attended outpatient appts (6m)** |  | Numeric | HES |  |  |  |  | x |  |  | x |  |  | 2 |
| **Number of DNA & cancelled outpatient appts (6m)** |  | Numeric | HES |  |  |  |  | x |  |  | x |  | x | 3 |
| **Acute Kidney Injury** |  | Binary | HES | x | x | x | x |  | x | x | x |  | x | 8 |
| **Agranulocytosis** |  | Binary | HES |  | x | x |  |  | x | x |  |  |  | 4 |
| **Alcohol and other substance misuse** |  | Binary | HES | x | x | x |  | x |  | x |  |  | x | 6 |
| **Anaemia** |  | Binary | HES | x | x | x | x | x | x | x | x | x | x | 10 |
| **Angina** |  | Binary | HES | x | x | x | x | x | x | x | x |  | x | 9 |
| **Anorectal fissure, fistula, prolapse** |  | Binary | HES |  | x | x |  |  |  | x |  |  | x | 4 |
| **Anxiety disorders** |  | Binary | HES | x | x | x | x | x | x | x | x |  | x | 9 |
| **Appendicitis** |  | Binary | HES |  | x | x |  |  |  | x | x |  | x | 5 |
| **Arterial disease** |  | Binary | HES | x | x | x |  |  |  | x |  |  | x | 5 |
| **Asthma** |  | Binary | HES | x | x | x | x | x | x | x | x | x | x | 10 |
| **Atrial fibrillation** |  | Binary | HES | x | x | x | x | x | x | x | x | x | x | 10 |
| **Bacterial disease** |  | Binary | HES | x | x | x | x | x | x | x | x | x | x | 10 |
| **Cardiomyopathy** |  | Binary | HES |  |  | x |  |  |  |  |  |  |  | 1 |
| **Cerebrovascular disease** |  | Binary | HES | x | x | x | x | x |  | x |  |  | x | 7 |
| **Chronic ishaemic heart disease** |  | Binary | HES | x | x | x | x | x | x | x | x | x | x | 10 |
| **Chronic kidney disease** |  | Binary | HES | x | x | x | x |  |  | x | x | x | x | 8 |
| **Coeliac disease** |  | Binary | HES |  | x | x |  |  |  |  |  |  |  | 2 |
| **COPD** |  | Binary | HES | x | x | x | x | x |  | x | x |  | x | 8 |
| **Depression** |  | Binary | HES | x | x | x | x | x | x | x | x | x | x | 10 |
| **Diabetes** |  | Binary | HES | x | x | x | x | x | x | x | x | x | x | 10 |
| **Diverticular disease of intestine (acute and chronic)** |  | Binary | HES | x | x | x | x | x | x | x | x | x | x | 10 |
| **Dorsopathy** |  | Binary | HES | x | x | x | x | x | x | x | x | x | x | 10 |
| **Ear and upper respiratory tract infection** |  | Binary | HES |  | x | x | x | x | x | x |  |  | x | 7 |
| **Enthesopathy, fibromatosis and synovial disorder** |  | Binary | HES | x | x | x | x |  |  | x | x | x | x | 8 |
| **Epilepsy** |  | Binary | HES |  | x | x |  | x |  | x |  |  | x | 5 |
| **Eye disease** |  | Binary | HES | x | x | x | x | x | x | x | x | x | x | 10 |
| **Female genital tract disorder** |  | Binary | HES | x | x | x | x | x | x | x | x |  | x | 9 |
| **Female infertility** |  | Binary | HES |  | x |  |  |  |  |  |  |  |  | 1 |
| **Fracture of hip and wrist** |  | Binary | HES |  | x | x | x |  |  | x |  |  |  | 4 |
| **Gastritis and duodenitis** |  | Binary | HES | x | x | x | x | x | x | x | x | x | x | 10 |
| **Hearing loss** |  | Binary | HES | x | x | x | x |  |  | x |  |  | x | 6 |
| **Heart block** |  | Binary | HES | x | x | x | x |  |  | x |  |  | x | 6 |
| **Heart failure** |  | Binary | HES | x | x | x | x |  |  | x |  |  | x | 6 |
| **Hernia** |  | Binary | HES | x | x | x | x | x | x | x | x | x | x | 10 |
| **Hyperparathyroidism** |  | Binary | HES |  | x |  |  |  |  |  |  |  |  | 1 |
| **Hyperplasia of prostate** |  | Binary | HES | x |  | x |  | x |  | x |  | x | x | 6 |
| **Hypertension** |  | Binary | HES | x | x | x | x | x | x | x | x | x | x | 10 |
| **Hypo or hyperthyroidism** |  | Binary | HES | x | x | x | x | x | x | x | x | x | x | 10 |
| **Infection of male genital system** |  | Binary | HES |  |  | x |  |  |  | x |  |  | x | 3 |
| **Infection of skin and subcutaneus tissues** |  | Binary | HES | x | x | x | x | x | x | x | x | x | x | 10 |
| **Inflammatory bowel disease** |  | Binary | HES |  | x | x | x |  |  | x |  |  | x | 5 |
| **Irritable bowel syndrome** |  | Binary | HES |  | x | x | x |  |  | x | x |  | x | 6 |
| **Liver disease** |  | Binary | HES | x | x | x | x |  | x | x |  |  | x | 7 |
| **Menstruation disorder** |  | Binary | HES |  | x | x | x | x |  | x | x |  | x | 7 |
| **Migraine** |  | Binary | HES |  | x | x |  | x |  | x | x |  | x | 6 |
| **Mycosis** |  | Binary | HES |  | x | x |  |  | x | x |  |  | x | 5 |
| **Myocardial infarction** |  | Binary | HES | x | x | x |  |  |  | x |  | x | x | 6 |
| **Nerve disorder** |  | Binary | HES | x | x | x | x | x | x | x | x | x | x | 10 |
| **Nervous system infection** |  | Binary | HES |  |  | x |  |  |  |  |  |  |  | 1 |
| **Neurodegenerative disease** |  | Binary | HES |  | x | x |  |  |  | x |  |  | x | 4 |
| **Nonrheumatic valve disorder** |  | Binary | HES | x | x | x | x |  |  | x |  |  | x | 6 |
| **Obesity** |  | Binary | HES | x | x | x | x | x | x | x | x | x | x | 10 |
| **Oesophagus disease** |  | Binary | HES | x | x | x | x | x | x | x | x | x | x | 10 |
| **Osteoarthritis (excl spine)** |  | Binary | HES | x | x | x | x | x | x | x | x | x | x | 10 |
| **Osteoporosis** |  | Binary | HES | x | x | x | x |  |  | x | x |  | x | 7 |
| **Other arthropathy** |  | Binary | HES | x |  | x |  | x |  | x |  | x | x | 6 |
| **Other heart rhythm disorder** |  | Binary | HES |  | x | x |  |  |  | x |  |  | x | 4 |
| **Other infection** |  | Binary | HES | x | x | x | x | x | x | x | x | x | x | 10 |
| **Other mental and behavioural disorder** |  | Binary | HES |  | x | x | x | x |  | x |  |  | x | 6 |
| **Other urinary system disease** |  | Binary | HES | x | x | x | x |  |  | x | x |  | x | 7 |
| **Pancreatitis and gallbladder disease** |  | Binary | HES | x | x | x | x | x | x | x | x | x | x | 10 |
| **Peptic ulcer disease** |  | Binary | HES | x | x | x | x |  |  | x |  |  | x | 6 |
| **Peritonitis** |  | Binary | HES |  | x | x |  |  |  | x |  |  | x | 4 |
| **Pleural disease** |  | Binary | HES | x | x | x | x |  | x | x | x |  | x | 8 |
| **Pulmonary disease** |  | Binary | HES | x | x | x | x | x | x | x | x | x | x | 10 |
| **Pulmonary embolism and hypertension** |  | Binary | HES |  | x | x | x |  |  | x | x |  | x | 6 |
| **Renal tubulo-interstitial disease** |  | Binary | HES | x | x | x | x |  |  | x | x |  | x | 7 |
| **Respiratory failure** |  | Binary | HES |  |  | x |  |  |  | x |  |  | x | 3 |
| **Rheumatic fever and valve disease** |  | Binary | HES | x | x | x |  |  |  | x |  |  | x | 5 |
| **Rheumatoid arthritis** |  | Binary | HES |  | x | x |  |  |  | x | x |  | x | 5 |
| **Rhinitis, sinusitis and nasal polyp** |  | Binary | HES |  | x | x | x | x |  | x |  |  | x | 6 |
| **Secondary polycythaemia** |  | Binary | HES |  |  |  |  |  |  |  |  |  | x | 1 |
| **Septicaemia** |  | Binary | HES |  | x | x | x |  | x | x |  |  | x | 6 |
| **Skin disease** |  | Binary | HES | x | x | x | x | x | x | x | x | x | x | 10 |
| **Sleep apnoea** |  | Binary | HES | x | x | x | x | x |  | x |  |  | x | 7 |
| **Spleen disease** |  | Binary | HES |  |  |  |  |  | x | x |  |  | x | 3 |
| **Systemic connective tissue disorder** |  | Binary | HES |  | x | x |  |  |  | x |  |  | x | 4 |
| **Thrombophilia and thrombocytopenia** |  | Binary | HES |  |  | x |  |  | x | x |  |  | x | 4 |
| **Urinary Tract Infections** |  | Binary | HES | x | x | x | x | x | x | x | x |  | x | 9 |
| **Urolithiasis** |  | Binary | HES | x | x | x | x |  |  | x |  | x | x | 7 |
| **Venous thromboembolic disease (Excl PE)** |  | Binary | HES |  | x | x |  |  |  | x |  |  | x | 4 |
| **Viral diseases (excl chronic hepatitis/HIV)** |  | Binary | HES | x | x | x | x | x | x | x |  |  | x | 8 |
| **Volvulus** |  | Binary | HES |  |  | x |  |  |  |  |  |  |  | 1 |
| **Multimorbidity** |  | Numeric | HES | x | x | x | x | x | x | x | x | x | x | 10 |
| **APC_germ** |  | Binary | Genomics England |  |  |  |  |  |  |  |  |  |  | 0 |
| **ATM_germ** |  | Binary | Genomics England |  |  |  |  |  |  |  |  |  |  | 0 |
| **BAP1_germ** |  | Binary | Genomics England |  |  |  |  |  |  |  |  |  |  | 0 |
| **BLM_germ** |  | Binary | Genomics England |  |  |  |  |  |  |  |  |  |  | 0 |
| **BRCA1_germ** |  | Binary | Genomics England |  | x |  |  |  |  |  | x |  |  | 2 |
| **BRCA2_germ** |  | Binary | Genomics England |  | x |  |  |  |  |  | x |  |  | 2 |
| **BRIP1_germ** |  | Binary | Genomics England |  |  |  |  |  |  |  |  |  |  | 0 |
| **CDKN2A_germ** |  | Binary | Genomics England |  |  |  |  |  |  |  |  |  |  | 0 |
| **FANCA_germ** |  | Binary | Genomics England |  |  |  |  |  |  |  |  |  |  | 0 |
| **FLCN_germ** |  | Binary | Genomics England |  |  |  |  |  |  |  |  |  |  | 0 |
| **GBA_germ** |  | Binary | Genomics England |  |  |  |  |  |  |  |  |  |  | 0 |
| **MLH1_germ** |  | Binary | Genomics England |  |  |  |  |  |  |  |  |  |  | 0 |
| **MSH2_germ** |  | Binary | Genomics England |  |  |  |  |  |  |  |  |  |  | 0 |
| **MSH6_germ** |  | Binary | Genomics England |  |  | x | x |  |  |  |  |  |  | 2 |
| **NF1_germ** |  | Binary | Genomics England |  |  |  |  |  |  |  |  |  |  | 0 |
| **NRAS_germ** |  | Binary | Genomics England |  |  |  |  |  |  |  |  |  |  | 0 |
| **PALB2_germ** |  | Binary | Genomics England |  | x |  |  |  |  |  |  |  |  | 1 |
| **PMS2_germ** |  | Binary | Genomics England |  |  |  |  |  |  |  |  |  |  | 0 |
| **PTEN_germ** |  | Binary | Genomics England |  |  |  |  |  |  |  |  |  |  | 0 |
| **RB1_germ** |  | Binary | Genomics England |  |  |  |  |  |  |  |  |  |  | 0 |
| **RUNX1_germ** |  | Binary | Genomics England |  |  |  |  |  |  |  |  |  |  | 0 |
| **SDHA_germ** |  | Binary | Genomics England |  |  |  |  |  |  |  |  |  |  | 0 |
| **TP53_germ** |  | Binary | Genomics England |  |  |  |  |  |  |  |  |  |  | 0 |
| **TSC1_germ** |  | Binary | Genomics England |  |  |  |  |  |  |  |  |  |  | 0 |
| **VHL_germ** |  | Binary | Genomics England |  |  |  |  |  |  |  |  |  |  | 0 |
| **WRN_germ** |  | Binary | Genomics England |  |  |  |  |  |  |  |  |  |  | 0 |
| **ABCA12** |  | Binary | Genomics England |  |  | x | x |  |  |  |  |  |  | 2 |
| **ABCA4** |  | Binary | Genomics England |  |  | x | x |  |  |  |  |  |  | 2 |
| **ABCB4** |  | Binary | Genomics England |  |  | x | x |  |  |  |  |  |  | 2 |
| **ABCC6** |  | Binary | Genomics England |  |  | x |  |  |  |  |  |  |  | 1 |
| **ABCC8** |  | Binary | Genomics England |  |  | x |  |  |  |  |  |  |  | 1 |
| **ACAN** |  | Binary | Genomics England |  |  | x |  |  |  |  |  |  |  | 1 |
| **ACVR1** |  | Binary | Genomics England |  |  |  | x |  |  |  |  |  |  | 1 |
| **ADAMTSL4** |  | Binary | Genomics England |  |  | x |  |  |  |  |  |  |  | 1 |
| **ADGRV1** |  | Binary | Genomics England |  |  | x |  |  |  |  |  |  |  | 1 |
| **ADNP** |  | Binary | Genomics England |  |  | x |  |  |  |  |  |  |  | 1 |
| **AGL** |  | Binary | Genomics England |  |  | x | x |  |  |  |  |  |  | 2 |
| **AGRN** |  | Binary | Genomics England |  |  | x | x |  |  |  |  |  |  | 2 |
| **AGT** |  | Binary | Genomics England |  |  | x |  |  |  |  |  |  |  | 1 |
| **AGXT** |  | Binary | Genomics England |  |  | x | x |  |  |  |  |  |  | 2 |
| **AHI1** |  | Binary | Genomics England |  |  | x | x |  |  |  |  |  |  | 2 |
| **ALG6** |  | Binary | Genomics England |  |  | x |  |  |  |  |  |  |  | 1 |
| **ALMS1** |  | Binary | Genomics England |  |  | x | x |  |  |  |  |  |  | 2 |
| **ALOX12B** |  | Binary | Genomics England |  |  | x |  |  |  |  |  |  |  | 1 |
| **ALPL** |  | Binary | Genomics England |  |  | x |  |  |  |  |  |  |  | 1 |
| **AMER1** |  | Binary | Genomics England |  |  | x |  |  |  |  |  |  |  | 1 |
| **ANKRD11** |  | Binary | Genomics England |  |  | x | x |  |  |  |  |  |  | 2 |
| **ANO5** |  | Binary | Genomics England |  |  | x |  |  |  |  |  |  |  | 1 |
| **ANTXR2** |  | Binary | Genomics England |  |  | x | x |  |  |  |  |  |  | 2 |
| **AP1S1** |  | Binary | Genomics England |  |  | x |  |  |  |  |  |  |  | 1 |
| **APC** |  | Binary | Genomics England |  |  | x | x |  |  |  |  |  |  | 2 |
| **AR** |  | Binary | Genomics England |  |  | x |  |  |  |  |  |  |  | 1 |
| **ARID1A** |  | Binary | Genomics England |  |  | x | x |  |  |  |  |  |  | 2 |
| **ARSA** |  | Binary | Genomics England |  |  | x |  |  |  |  |  |  |  | 1 |
| **ASL** |  | Binary | Genomics England |  |  | x |  |  |  |  |  |  |  | 1 |
| **ASPM** |  | Binary | Genomics England |  |  | x | x |  |  |  |  |  |  | 2 |
| **ASXL1** |  | Binary | Genomics England |  |  | x |  |  |  |  |  |  |  | 1 |
| **ATM** |  | Binary | Genomics England |  |  | x | x |  |  | x |  |  |  | 3 |
| **ATP13A3** |  | Binary | Genomics England |  |  | x |  |  |  |  |  |  |  | 1 |
| **ATP2A1** |  | Binary | Genomics England |  |  | x | x |  |  |  |  |  |  | 2 |
| **ATP6V1B1** |  | Binary | Genomics England |  |  | x | x |  |  |  |  |  |  | 2 |
| **ATP7B** |  | Binary | Genomics England |  |  | x |  |  |  |  |  |  |  | 1 |
| **ATP8B1** |  | Binary | Genomics England |  |  | x | x |  |  |  |  |  |  | 2 |
| **ATR** |  | Binary | Genomics England |  | x | x | x |  |  |  |  |  |  | 3 |
| **AXIN2** |  | Binary | Genomics England |  |  | x | x |  |  |  |  |  |  | 2 |
| **B3GALT6** |  | Binary | Genomics England |  |  | x |  |  |  |  |  |  |  | 1 |
| **B4GALNT1** |  | Binary | Genomics England |  |  | x |  |  |  |  |  |  |  | 1 |
| **BAP1** |  | Binary | Genomics England |  |  |  |  |  |  |  |  |  | x | 1 |
| **BAX** |  | Binary | Genomics England |  |  | x | x |  |  |  |  |  |  | 2 |
| **BCKDHA** |  | Binary | Genomics England |  |  | x | x |  |  |  |  |  |  | 2 |
| **BCL10** |  | Binary | Genomics England |  |  | x |  |  |  |  |  |  |  | 1 |
| **BCL11A** |  | Binary | Genomics England |  |  | x | x |  |  |  |  |  |  | 2 |
| **BCOR** |  | Binary | Genomics England |  |  | x |  |  |  |  |  |  |  | 1 |
| **BLM** |  | Binary | Genomics England |  |  | x | x |  |  |  |  |  |  | 2 |
| **BMPR1A** |  | Binary | Genomics England |  |  | x |  |  |  |  |  |  |  | 1 |
| **BMPR2** |  | Binary | Genomics England |  |  | x |  |  |  |  |  |  |  | 1 |
| **BPTF** |  | Binary | Genomics England |  |  | x |  |  |  |  |  |  |  | 1 |
| **BRAF** |  | Binary | Genomics England |  |  | x |  |  |  | x |  |  |  | 2 |
| **BRCA1** |  | Binary | Genomics England |  | x | x | x |  |  |  |  |  |  | 3 |
| **BRCA2** |  | Binary | Genomics England |  | x | x | x |  |  |  |  |  |  | 3 |
| **BRIP1** |  | Binary | Genomics England |  |  |  |  |  |  |  |  |  |  | 0 |
| **CAPN3** |  | Binary | Genomics England |  |  |  | x |  |  |  |  |  |  | 1 |
| **CASR** |  | Binary | Genomics England |  |  | x |  |  |  |  |  |  |  | 1 |
| **CC2D2A** |  | Binary | Genomics England |  |  | x | x |  |  |  |  |  |  | 2 |
| **CD3G** |  | Binary | Genomics England |  |  | x |  |  |  |  |  |  |  | 1 |
| **CDH1** |  | Binary | Genomics England |  | x | x |  |  |  |  |  |  |  | 2 |
| **CDKL5** |  | Binary | Genomics England |  |  | x |  |  |  |  |  |  |  | 1 |
| **CDKN2A** |  | Binary | Genomics England |  |  | x |  |  |  | x |  |  |  | 2 |
| **CEP290** |  | Binary | Genomics England |  | x | x | x |  |  |  |  |  |  | 3 |
| **CFTR** |  | Binary | Genomics England |  |  | x | x |  |  |  |  |  |  | 2 |
| **CHD2** |  | Binary | Genomics England |  |  | x | x |  |  |  |  |  |  | 2 |
| **CHD7** |  | Binary | Genomics England |  |  | x | x |  |  |  |  |  |  | 2 |
| **CHEK2** |  | Binary | Genomics England |  |  | x |  |  |  |  |  |  |  | 1 |
| **CHRNA3** |  | Binary | Genomics England |  | x | x |  |  |  |  |  |  |  | 2 |
| **CIITA** |  | Binary | Genomics England |  |  | x |  |  |  |  |  |  |  | 1 |
| **CLCN1** |  | Binary | Genomics England |  |  |  | x |  |  |  |  |  |  | 1 |
| **CNGB3** |  | Binary | Genomics England |  |  | x |  |  |  |  |  |  |  | 1 |
| **COL12A1** |  | Binary | Genomics England |  |  | x |  |  |  |  |  |  |  | 1 |
| **COL1A1** |  | Binary | Genomics England |  |  | x | x |  |  |  |  |  |  | 2 |
| **COL1A2** |  | Binary | Genomics England |  |  | x |  |  |  |  |  |  |  | 1 |
| **COL2A1** |  | Binary | Genomics England |  |  | x |  |  |  |  |  |  |  | 1 |
| **COL3A1** |  | Binary | Genomics England |  |  | x |  |  |  |  |  |  |  | 1 |
| **COL4A1** |  | Binary | Genomics England |  |  | x |  |  |  |  |  |  |  | 1 |
| **COL4A5** |  | Binary | Genomics England |  |  |  | x |  |  |  |  |  |  | 1 |
| **COL5A1** |  | Binary | Genomics England |  |  | x | x |  |  |  |  |  |  | 2 |
| **COL7A1** |  | Binary | Genomics England |  |  | x | x |  |  |  |  |  |  | 2 |
| **CPS1** |  | Binary | Genomics England |  |  |  | x |  |  |  |  |  |  | 1 |
| **CRB1** |  | Binary | Genomics England |  |  |  | x |  |  |  |  |  |  | 1 |
| **CREBBP** |  | Binary | Genomics England |  |  | x | x |  |  |  |  |  |  | 2 |
| **CSF3R** |  | Binary | Genomics England |  |  | x |  |  |  |  |  |  |  | 1 |
| **CTCF** |  | Binary | Genomics England |  |  | x | x |  |  |  |  |  |  | 2 |
| **CTNNB1** |  | Binary | Genomics England |  |  | x | x |  |  | x | x |  |  | 4 |
| **CYP21A2** |  | Binary | Genomics England |  |  | x |  |  |  |  |  |  |  | 1 |
| **CYP7B1** |  | Binary | Genomics England |  |  | x |  |  |  |  |  |  |  | 1 |
| **DCX** |  | Binary | Genomics England |  |  | x |  |  |  |  |  |  |  | 1 |
| **DDX11** |  | Binary | Genomics England |  | x | x |  |  |  |  |  |  |  | 2 |
| **DDX3X** |  | Binary | Genomics England |  |  | x | x |  |  |  |  |  |  | 2 |
| **DGKE** |  | Binary | Genomics England |  |  | x |  |  |  |  |  |  |  | 1 |
| **DHCR7** |  | Binary | Genomics England |  |  | x |  |  |  |  |  |  |  | 1 |
| **DICER1** |  | Binary | Genomics England |  |  | x | x |  |  |  |  |  |  | 2 |
| **DLD** |  | Binary | Genomics England |  |  | x |  |  |  |  |  |  |  | 1 |
| **DLL1** |  | Binary | Genomics England |  |  | x |  |  |  |  |  |  |  | 1 |
| **DMD** |  | Binary | Genomics England |  |  | x | x |  |  |  |  |  |  | 2 |
| **DNAAF4** |  | Binary | Genomics England |  |  | x | x |  |  |  |  |  |  | 2 |
| **DNAH11** |  | Binary | Genomics England |  |  | x |  |  |  |  |  |  |  | 1 |
| **DNAH5** |  | Binary | Genomics England |  |  | x | x |  |  |  |  |  |  | 2 |
| **DOCK8** |  | Binary | Genomics England |  |  | x | x |  |  |  |  |  |  | 2 |
| **DOK7** |  | Binary | Genomics England |  |  | x | x |  |  |  |  |  |  | 2 |
| **DSP** |  | Binary | Genomics England |  |  | x |  |  |  |  |  |  |  | 1 |
| **DYNC1H1** |  | Binary | Genomics England |  |  | x |  |  |  |  |  |  |  | 1 |
| **DYNC2H1** |  | Binary | Genomics England |  |  | x | x |  |  |  |  |  |  | 2 |
| **EGFR** |  | Binary | Genomics England |  |  |  |  | x |  | x |  |  |  | 2 |
| **EIF2B3** |  | Binary | Genomics England |  |  | x | x |  |  |  |  |  |  | 2 |
| **ENAM** |  | Binary | Genomics England |  |  | x |  |  |  |  |  |  |  | 1 |
| **ERBB2** |  | Binary | Genomics England | x | x | x | x |  |  |  |  |  |  | 4 |
| **ERBB3** |  | Binary | Genomics England |  |  | x | x |  |  |  |  |  |  | 2 |
| **ESCO2** |  | Binary | Genomics England |  |  | x | x |  |  |  |  |  |  | 2 |
| **EXT1** |  | Binary | Genomics England |  |  | x |  |  |  |  |  |  |  | 1 |
| **EYS** |  | Binary | Genomics England |  |  | x | x |  |  |  |  |  |  | 2 |
| **F8** |  | Binary | Genomics England |  | x | x | x |  |  |  |  |  |  | 3 |
| **FAH** |  | Binary | Genomics England |  |  | x |  |  |  |  |  |  |  | 1 |
| **FANCA** |  | Binary | Genomics England |  |  | x |  |  |  |  |  |  |  | 1 |
| **FANCE** |  | Binary | Genomics England |  |  | x |  |  |  |  |  |  |  | 1 |
| **FBN1** |  | Binary | Genomics England |  |  | x | x |  |  |  |  |  |  | 2 |
| **FBXL3** |  | Binary | Genomics England |  | x | x | x |  |  |  |  |  | x | 4 |
| **FBXW7** |  | Binary | Genomics England |  |  | x | x |  |  |  |  |  |  | 2 |
| **FGD1** |  | Binary | Genomics England |  |  | x | x |  |  |  |  |  |  | 2 |
| **FGFR2** |  | Binary | Genomics England |  |  |  | x |  |  |  |  |  |  | 1 |
| **FGFR3** |  | Binary | Genomics England | x |  |  |  |  |  |  |  |  |  | 1 |
| **FKBP10** |  | Binary | Genomics England |  |  | x | x |  |  |  |  |  |  | 2 |
| **FKTN** |  | Binary | Genomics England |  |  | x |  |  |  |  |  |  |  | 1 |
| **FLCN** |  | Binary | Genomics England |  |  | x | x |  |  |  |  |  |  | 2 |
| **FLG** |  | Binary | Genomics England |  |  | x |  |  |  |  |  |  |  | 1 |
| **FLNA** |  | Binary | Genomics England |  |  | x |  |  |  |  |  |  |  | 1 |
| **FLNB** |  | Binary | Genomics England |  |  | x |  |  |  |  |  |  |  | 1 |
| **FLT4** |  | Binary | Genomics England |  |  | x |  |  |  |  |  |  |  | 1 |
| **FOXG1** |  | Binary | Genomics England |  |  | x | x |  |  |  |  |  |  | 2 |
| **GAA** |  | Binary | Genomics England |  |  | x |  |  |  |  |  |  |  | 1 |
| **GALC** |  | Binary | Genomics England |  |  | x |  |  |  |  |  |  |  | 1 |
| **GAMT** |  | Binary | Genomics England |  |  | x |  |  |  |  |  |  |  | 1 |
| **GATA2** |  | Binary | Genomics England |  |  | x |  |  |  |  |  |  |  | 1 |
| **GATA3** |  | Binary | Genomics England |  | x | x |  |  |  |  |  |  |  | 2 |
| **GBA** |  | Binary | Genomics England |  |  | x |  |  |  |  |  |  |  | 1 |
| **GCDH** |  | Binary | Genomics England |  |  | x | x |  |  |  |  |  |  | 2 |
| **GCK** |  | Binary | Genomics England |  |  | x |  |  |  |  |  |  |  | 1 |
| **GDF5** |  | Binary | Genomics England |  |  | x |  |  |  |  |  |  |  | 1 |
| **GJC2** |  | Binary | Genomics England |  |  | x |  |  |  |  |  |  |  | 1 |
| **GLB1** |  | Binary | Genomics England |  |  | x |  |  |  |  |  |  |  | 1 |
| **GLDC** |  | Binary | Genomics England |  |  | x | x |  |  |  |  |  |  | 2 |
| **GNPTAB** |  | Binary | Genomics England |  |  | x | x |  |  |  |  |  |  | 2 |
| **GRIN2A** |  | Binary | Genomics England |  |  | x |  |  |  |  |  |  |  | 1 |
| **GRIN2B** |  | Binary | Genomics England |  |  | x |  |  |  |  |  |  |  | 1 |
| **GUCY2D** |  | Binary | Genomics England |  |  | x |  |  |  |  |  |  |  | 1 |
| **HEXA** |  | Binary | Genomics England |  |  | x |  |  |  |  |  |  |  | 1 |
| **HNF1A** |  | Binary | Genomics England |  |  | x | x |  |  |  |  |  |  | 2 |
| **HNF1B** |  | Binary | Genomics England |  |  | x |  |  |  |  |  |  |  | 1 |
| **HPS1** |  | Binary | Genomics England |  |  | x | x |  |  |  |  |  |  | 2 |
| **HPSE2** |  | Binary | Genomics England |  |  | x |  |  |  |  |  |  |  | 1 |
| **HRAS** |  | Binary | Genomics England | x |  |  |  |  |  |  |  |  |  | 1 |
| **HSPG2** |  | Binary | Genomics England |  |  | x |  |  |  |  |  |  |  | 1 |
| **IDH1** |  | Binary | Genomics England |  |  | x |  | x |  |  |  |  |  | 2 |
| **IDH2** |  | Binary | Genomics England |  |  |  |  |  | x |  |  |  |  | 1 |
| **IDS** |  | Binary | Genomics England |  |  |  | x |  |  |  |  |  |  | 1 |
| **IQSEC2** |  | Binary | Genomics England |  |  | x | x |  |  |  |  |  |  | 2 |
| **IRS4** |  | Binary | Genomics England |  |  | x | x |  |  |  |  |  |  | 2 |
| **JAK3** |  | Binary | Genomics England |  |  | x |  |  |  |  |  |  |  | 1 |
| **KANSL1** |  | Binary | Genomics England |  |  | x | x |  |  |  |  |  |  | 2 |
| **KCNC1** |  | Binary | Genomics England |  |  | x |  |  |  |  |  |  |  | 1 |
| **KCNH2** |  | Binary | Genomics England |  |  | x | x |  |  |  |  |  |  | 2 |
| **KCNQ1** |  | Binary | Genomics England |  |  | x | x |  |  |  |  |  |  | 2 |
| **KCNQ2** |  | Binary | Genomics England |  |  | x |  |  |  |  |  |  |  | 1 |
| **KMT2A** |  | Binary | Genomics England |  |  | x | x |  |  |  |  |  |  | 2 |
| **KMT2B** |  | Binary | Genomics England |  |  | x | x |  |  |  |  |  |  | 2 |
| **KMT2C** |  | Binary | Genomics England |  |  | x | x |  |  |  |  |  |  | 2 |
| **KMT2D** |  | Binary | Genomics England | x |  | x | x |  |  | x |  |  |  | 4 |
| **KRAS** |  | Binary | Genomics England | x |  | x | x |  |  | x | x |  |  | 5 |
| **LAMA2** |  | Binary | Genomics England |  |  | x | x |  |  |  |  |  |  | 2 |
| **LAT** |  | Binary | Genomics England |  |  | x |  |  |  |  |  |  |  | 1 |
| **LDLR** |  | Binary | Genomics England |  |  | x | x |  |  |  |  |  |  | 2 |
| **LDLRAP1** |  | Binary | Genomics England |  |  | x |  |  |  |  |  |  |  | 1 |
| **LRP2** |  | Binary | Genomics England |  |  | x | x |  |  |  |  |  |  | 2 |
| **LTBP3** |  | Binary | Genomics England |  |  | x |  |  |  |  |  |  |  | 1 |
| **LZTR1** |  | Binary | Genomics England |  |  | x | x |  |  |  |  |  |  | 2 |
| **MAGEL2** |  | Binary | Genomics England |  |  | x | x |  |  |  |  |  |  | 2 |
| **MAP2K1** |  | Binary | Genomics England |  |  | x |  |  |  |  |  |  |  | 1 |
| **MAP3K6** |  | Binary | Genomics England |  |  | x |  |  |  |  |  |  |  | 1 |
| **MCPH1** |  | Binary | Genomics England |  |  | x | x |  |  |  |  |  |  | 2 |
| **MED12** |  | Binary | Genomics England |  |  |  | x |  |  |  |  |  |  | 1 |
| **MFRP** |  | Binary | Genomics England |  |  | x | x |  |  |  |  |  |  | 2 |
| **MLH1** |  | Binary | Genomics England |  |  | x | x |  |  |  |  |  |  | 2 |
| **MSH2** |  | Binary | Genomics England |  | x | x | x |  |  |  |  |  |  | 3 |
| **MSH3** |  | Binary | Genomics England |  |  | x | x |  |  |  |  |  |  | 2 |
| **MSH6** |  | Binary | Genomics England |  |  | x | x |  |  |  |  |  |  | 2 |
| **MTM1** |  | Binary | Genomics England |  |  | x | x |  |  |  |  |  |  | 2 |
| **MTOR** |  | Binary | Genomics England |  |  | x | x |  |  |  |  |  | x | 3 |
| **MYBPC3** |  | Binary | Genomics England |  |  | x |  |  |  |  |  |  |  | 1 |
| **MYH7** |  | Binary | Genomics England |  |  | x |  |  |  |  |  |  |  | 1 |
| **MYO15A** |  | Binary | Genomics England |  |  | x |  |  |  |  |  |  |  | 1 |
| **MYO6** |  | Binary | Genomics England |  | x | x |  |  |  |  |  |  |  | 2 |
| **MYO7A** |  | Binary | Genomics England |  |  | x | x |  |  |  |  |  |  | 2 |
| **MYRF** |  | Binary | Genomics England |  |  | x |  |  |  |  |  |  |  | 1 |
| **NALCN** |  | Binary | Genomics England |  |  | x |  |  |  |  |  |  |  | 1 |
| **NBN** |  | Binary | Genomics England |  |  | x | x |  |  |  |  |  |  | 2 |
| **NCF1** |  | Binary | Genomics England |  | x | x |  |  |  |  |  |  |  | 2 |
| **NCSTN** |  | Binary | Genomics England |  |  | x |  |  |  |  |  |  |  | 1 |
| **NEB** |  | Binary | Genomics England |  |  | x | x |  |  |  |  |  |  | 2 |
| **NEK1** |  | Binary | Genomics England |  |  | x |  |  |  |  |  |  |  | 1 |
| **NF1** |  | Binary | Genomics England |  | x | x | x | x |  | x |  |  |  | 5 |
| **NFE2L2** |  | Binary | Genomics England |  |  |  |  |  |  | x |  |  |  | 1 |
| **NHLRC1** |  | Binary | Genomics England |  |  | x |  |  |  |  |  |  |  | 1 |
| **NIPBL** |  | Binary | Genomics England |  |  | x | x |  |  |  |  |  |  | 2 |
| **NKX62** |  | Binary | Genomics England |  |  | x |  |  |  |  |  |  |  | 1 |
| **NOBOX** |  | Binary | Genomics England |  |  | x |  |  |  |  |  |  |  | 1 |
| **NPHP1** |  | Binary | Genomics England |  |  | x |  |  |  |  |  |  |  | 1 |
| **NPHS1** |  | Binary | Genomics England |  |  | x |  |  |  |  |  |  |  | 1 |
| **NPM1** |  | Binary | Genomics England |  |  |  |  |  | x |  |  |  |  | 1 |
| **NR4A2** |  | Binary | Genomics England |  |  | x |  |  |  |  |  |  |  | 1 |
| **NRAS** |  | Binary | Genomics England |  |  | x | x |  | x |  |  |  |  | 3 |
| **NSD1** |  | Binary | Genomics England |  |  |  | x |  |  |  |  |  |  | 1 |
| **NSD2** |  | Binary | Genomics England |  |  | x | x |  |  |  |  |  |  | 2 |
| **OFD1** |  | Binary | Genomics England |  |  | x | x |  |  |  |  |  |  | 2 |
| **OPTN** |  | Binary | Genomics England |  |  | x |  |  |  |  |  |  |  | 1 |
| **OTC** |  | Binary | Genomics England |  |  |  | x |  |  |  |  |  |  | 1 |
| **PAFAH1B1** |  | Binary | Genomics England |  |  | x |  |  |  |  |  |  |  | 1 |
| **PAH** |  | Binary | Genomics England |  |  | x |  |  |  |  |  |  |  | 1 |
| **PALB2** |  | Binary | Genomics England |  |  | x |  |  |  |  |  |  |  | 1 |
| **PAX2** |  | Binary | Genomics England |  |  | x |  |  |  |  |  |  |  | 1 |
| **PCCA** |  | Binary | Genomics England |  |  | x |  |  |  |  |  |  |  | 1 |
| **PCDH15** |  | Binary | Genomics England |  |  | x |  |  |  |  |  |  |  | 1 |
| **PCDH19** |  | Binary | Genomics England |  |  | x | x |  |  |  |  |  |  | 2 |
| **PEX1** |  | Binary | Genomics England |  |  | x | x |  |  |  |  |  |  | 2 |
| **PIGO** |  | Binary | Genomics England |  |  | x | x |  |  |  |  |  |  | 2 |
| **PIK3CA** |  | Binary | Genomics England | x | x | x | x | x |  | x | x | x | x | 9 |
| **PIK3R1** |  | Binary | Genomics England |  |  | x | x |  |  |  |  |  |  | 2 |
| **PIK3R2** |  | Binary | Genomics England |  |  |  | x |  |  |  |  |  |  | 1 |
| **PKD1** |  | Binary | Genomics England |  |  | x |  |  |  |  |  |  |  | 1 |
| **PKD2** |  | Binary | Genomics England |  |  | x | x |  |  |  |  |  |  | 2 |
| **PKHD1** |  | Binary | Genomics England |  |  | x | x |  |  |  |  |  |  | 2 |
| **PMS2** |  | Binary | Genomics England |  |  | x |  |  |  |  |  |  |  | 1 |
| **PPM1D** |  | Binary | Genomics England |  |  | x |  |  |  |  |  |  |  | 1 |
| **PPP2R1A** |  | Binary | Genomics England |  |  | x | x |  |  |  | x |  |  | 3 |
| **PQBP1** |  | Binary | Genomics England |  |  | x |  |  |  |  |  |  |  | 1 |
| **PROM1** |  | Binary | Genomics England |  |  | x | x |  |  |  |  |  |  | 2 |
| **PRRT2** |  | Binary | Genomics England |  | x | x | x |  |  |  |  |  |  | 3 |
| **PRSS56** |  | Binary | Genomics England |  |  | x |  |  |  |  |  |  |  | 1 |
| **PTCH1** |  | Binary | Genomics England |  |  | x |  |  |  |  |  |  |  | 1 |
| **PTEN** |  | Binary | Genomics England |  | x | x | x | x |  | x | x | x | x | 8 |
| **PTPN11** |  | Binary | Genomics England |  |  | x |  | x |  |  |  |  |  | 2 |
| **PXDN** |  | Binary | Genomics England |  |  | x | x |  |  |  |  |  |  | 2 |
| **RAD50** |  | Binary | Genomics England |  |  | x | x |  |  |  |  |  |  | 2 |
| **RAG1** |  | Binary | Genomics England |  |  | x | x |  |  |  |  |  |  | 2 |
| **RAI1** |  | Binary | Genomics England |  |  | x |  |  |  |  |  |  |  | 1 |
| **RASA1** |  | Binary | Genomics England |  |  |  | x |  |  |  |  |  |  | 1 |
| **RB1** |  | Binary | Genomics England | x | x | x | x | x |  | x |  |  |  | 6 |
| **RNF43** |  | Binary | Genomics England |  |  | x | x |  |  |  |  |  |  | 2 |
| **RP1** |  | Binary | Genomics England |  |  | x | x |  |  |  |  |  |  | 2 |
| **RPE65** |  | Binary | Genomics England |  |  | x |  |  |  |  |  |  |  | 1 |
| **RUNX1** |  | Binary | Genomics England |  | x | x |  |  |  |  |  |  |  | 2 |
| **RUNX2** |  | Binary | Genomics England |  |  | x |  |  |  |  |  |  |  | 1 |
| **RXRA** |  | Binary | Genomics England | x |  |  |  |  |  |  |  |  |  | 1 |
| **RYR1** |  | Binary | Genomics England |  |  | x | x |  |  |  |  |  |  | 2 |
| **RYR2** |  | Binary | Genomics England |  |  | x |  |  |  |  |  |  |  | 1 |
| **SATB2** |  | Binary | Genomics England |  |  | x |  |  |  |  |  |  |  | 1 |
| **SCN1A** |  | Binary | Genomics England |  |  | x |  |  |  |  |  |  |  | 1 |
| **SCN2A** |  | Binary | Genomics England |  |  | x | x |  |  |  |  |  |  | 2 |
| **SCN4A** |  | Binary | Genomics England |  |  | x |  |  |  |  |  |  |  | 1 |
| **SDHA** |  | Binary | Genomics England |  |  |  |  |  |  |  |  |  |  | 0 |
| **SERPINA1** |  | Binary | Genomics England |  |  | x |  |  |  |  |  |  |  | 1 |
| **SETD5** |  | Binary | Genomics England |  |  | x |  |  |  |  |  |  |  | 1 |
| **SF3B1** |  | Binary | Genomics England |  | x |  |  |  | x |  |  |  |  | 2 |
| **SF3B4** |  | Binary | Genomics England |  |  | x | x |  |  |  |  |  |  | 2 |
| **SIX3** |  | Binary | Genomics England |  |  | x |  |  |  |  |  |  |  | 1 |
| **SLC12A1** |  | Binary | Genomics England |  |  | x |  |  |  |  |  |  |  | 1 |
| **SLC26A2** |  | Binary | Genomics England |  |  | x |  |  |  |  |  |  |  | 1 |
| **SLC26A4** |  | Binary | Genomics England |  |  | x |  |  |  |  |  |  |  | 1 |
| **SLC4A11** |  | Binary | Genomics England |  |  | x |  |  |  |  |  |  |  | 1 |
| **SLCO2A1** |  | Binary | Genomics England |  |  | x |  |  |  |  |  |  |  | 1 |
| **SMAD3** |  | Binary | Genomics England |  |  | x |  |  |  |  |  |  |  | 1 |
| **SMAD4** |  | Binary | Genomics England |  |  | x |  |  |  | x |  |  |  | 2 |
| **SMARCA4** |  | Binary | Genomics England |  |  | x |  |  |  |  |  |  |  | 1 |
| **SMARCB1** |  | Binary | Genomics England |  |  | x |  |  |  |  |  |  |  | 1 |
| **SMC1A** |  | Binary | Genomics England |  |  |  | x |  |  |  |  |  |  | 1 |
| **SMPD1** |  | Binary | Genomics England |  | x | x |  |  |  |  |  |  |  | 2 |
| **SOX9** |  | Binary | Genomics England |  |  | x |  |  |  |  |  |  |  | 1 |
| **SPART** |  | Binary | Genomics England |  |  | x |  |  |  |  |  |  |  | 1 |
| **SPG11** |  | Binary | Genomics England |  |  | x |  |  |  |  |  |  |  | 1 |
| **SPINK5** |  | Binary | Genomics England |  | x | x | x |  |  |  |  |  |  | 3 |
| **SPOP** |  | Binary | Genomics England |  |  |  |  |  |  |  |  | x |  | 1 |
| **SPTA1** |  | Binary | Genomics England |  |  | x |  |  |  |  |  |  |  | 1 |
| **STAT5B** |  | Binary | Genomics England |  |  | x |  |  |  |  |  |  |  | 1 |
| **STK11** |  | Binary | Genomics England |  |  |  |  |  |  | x |  |  |  | 1 |
| **SUFU** |  | Binary | Genomics England |  |  | x |  |  |  |  |  |  |  | 1 |
| **SYNE1** |  | Binary | Genomics England |  |  | x | x |  |  |  |  |  |  | 2 |
| **SYNGAP1** |  | Binary | Genomics England |  |  | x |  |  |  |  |  |  |  | 1 |
| **TBCK** |  | Binary | Genomics England |  |  | x |  |  |  |  |  |  |  | 1 |
| **TBK1** |  | Binary | Genomics England |  |  | x |  |  |  |  |  |  |  | 1 |
| **TBX5** |  | Binary | Genomics England |  |  | x |  |  |  |  |  |  |  | 1 |
| **TP53** |  | Binary | Genomics England | x | x | x | x | x |  | x | x | x | x | 9 |
| **TPO** |  | Binary | Genomics England |  |  | x |  |  |  |  |  |  |  | 1 |
| **TRIOBP** |  | Binary | Genomics England |  |  | x |  |  |  |  |  |  |  | 1 |
| **TRIP11** |  | Binary | Genomics England |  |  | x |  |  |  |  |  |  |  | 1 |
| **TRNT1** |  | Binary | Genomics England |  |  | x |  |  |  |  |  |  |  | 1 |
| **TRPS1** |  | Binary | Genomics England |  |  | x |  |  |  |  |  |  |  | 1 |
| **TSC1** |  | Binary | Genomics England | x |  | x | x |  |  |  |  |  |  | 3 |
| **TSC2** |  | Binary | Genomics England |  |  | x |  |  |  |  |  |  |  | 1 |
| **TTN** |  | Binary | Genomics England |  | x | x | x |  |  |  |  |  |  | 3 |
| **TUBA1A** |  | Binary | Genomics England |  |  | x |  |  |  |  |  |  |  | 1 |
| **TUBB8** |  | Binary | Genomics England | x | x | x | x | x | x | x | x | x | x | 10 |
| **TYRP1** |  | Binary | Genomics England |  |  | x |  |  |  |  |  |  |  | 1 |
| **USH2A** |  | Binary | Genomics England |  |  | x | x |  |  | x |  |  |  | 3 |
| **USP9X** |  | Binary | Genomics England |  |  | x |  |  |  |  |  |  |  | 1 |
| **VHL** |  | Binary | Genomics England |  |  |  |  |  |  |  |  |  | x | 1 |
| **VWF** |  | Binary | Genomics England |  |  | x | x |  |  |  |  |  |  | 2 |
| **WDR19** |  | Binary | Genomics England |  |  | x |  |  |  |  |  |  |  | 1 |
| **WNT1** |  | Binary | Genomics England |  |  | x |  |  |  |  |  |  |  | 1 |
| **WRN** |  | Binary | Genomics England |  |  | x | x |  |  |  |  |  |  | 2 |
| **YY1** |  | Binary | Genomics England |  |  | x |  |  |  |  |  |  |  | 1 |
| **ZEB2** |  | Binary | Genomics England |  |  | x |  |  |  |  |  |  |  | 1 |
| **ZMPSTE24** |  | Binary | Genomics England |  |  | x |  |  |  |  |  |  |  | 1 |
| **ZNF292** |  | Binary | Genomics England |  |  | x | x |  |  |  |  |  |  | 2 |

**Supplementary File 2.** Result summary for all models (best model marked in bold)

**Bladder cancer**

| **Dataset** | **Cox Elastic Net regression** | | **Random Survival Forest** | | **Gradient Boosting Survival** | | **DeepSurv Neural Network** | |
| --- | --- | --- | --- | --- | --- | --- | --- | --- |
| **1^st^ set** | L1 ratio = 0.8 Alpha = 0.0247 Initial features = 99 Features selected = 29 | C-index (mean 5-fold cross validation) = 70.28% C-index (test data) = 60.93% | Max depth = 20 Max features = 0.2 Max samples = 0.5 Min samples leaf = 3 Min samples split = 2 Initial features = 99 Features selected = 24 | C-index (mean 5-fold cross validation) = 71.02% C-index (test data) = 63.30% | Max depth = 20 Max features = 0.2 Min samples leaf = 1  Min samples split = 10 Subsample = 0.5 Initial features = 99 Features selected = 20 | C-index (mean 5-fold cross validation) = 71.51% C-index (test data) = 62.28% |  |  |
| **2^nd^ set** | L1 ratio = 0.9 Alpha = 0.0153 Initial features = 99 Features selected = 32 | C-index (mean 5-fold cross validation) = 70.72% C-index (test data) = 62.15% | Max depth = 20 Max features = 0.2 Max samples = 0.5 Min samples leaf = 3 Min samples split = 2 Initial features = 99 Features selected = 24 | C-index (mean 5-fold cross validation) = 71.06% C-index (test data) = 63.46% | Max depth = 20 Max features = 0.8 Min samples leaf = 3 Min samples split = 10 Subsample = 0.5 Initial features = 99 Features selected = 20 | C-index (mean 5-fold cross validation) = 71.44% C-index (test data) = 61.75% | Num nodes = 64 Dropout = 0.50 Learning rate = 0.011 Batch size = 256 Number of epochs = 181 Initial features = 99 Features selected = 30 | C-index (mean 5-fold cross validation) = 83.56% C-index (test data) = 60.40% |
| **3^rd^ set** | L1 ratio = 0.7 Alpha = 0.0326 Initial features = 87 Features selected = 22 | C-index (mean 5-fold cross validation) = 70.42% C-index (test data) = 61.22% | Max depth = 20 Max features = 0.2 Max samples = 0.5 Min samples leaf = 3 Min samples split = 2 Initial features = 87 Features selected = 21 | C-index (mean 5-fold cross validation) = 71.23% C-index (test data) = 62.81% | **Max depth = 20 Max features = 0.5 Min samples leaf = 3 Min samples split = 2 Subsample = 0.5 Initial features = 87 Features selected = 19** | **C-index (mean 5-fold cross validation) = 71.28% C-index (test data) = 63.66%** |  |  |
| **4^th^ set** | L1 ratio = 0.9 Alpha = 0.0127 Initial features = 87 Features selected = 29 | C-index (mean 5-fold cross validation) = 70.38% C-index (test data) = 61.30% | Max depth = 20 Max features = 0.2 Max samples = 0.5 Min samples leaf = 3 Min samples split = 2 Initial features = 87 Features selected = 22 | C-index (mean 5-fold cross validation) = 71.70% C-index (test data) = 63.46% | Max depth = 20 Max features = 0.5 Min samples leaf = 3 Min samples split = 2 Subsample = 0.5 Initial features = 87 Features selected = 19 | C-index (mean 5-fold cross validation) = 71.10% C-index (test data) = 63.01% | Num nodes = 128 Dropout = 0.54 Learning rate = 0.010 Batch size = 128 Number of epochs = 126 Initial features = 87 Features selected = 25 | C-index (mean 5-fold cross validation) = 80.78% C-index (test data) = 59.32% |

**Breast cancer**

| **Dataset** | **Cox Elastic Net regression** | | **Random Survival Forest** | | **Gradient Boosting Survival** | | **DeepSurv Neural Network** | |
| --- | --- | --- | --- | --- | --- | --- | --- | --- |
| **1^st^ set** | L1 ratio = 1 Alpha = 0.0090 Initial features = 178 Features selected = 28 | C-index (mean 5-fold cross validation) = 71.65% C-index (test data) = 70.47% | Max depth = 50 Max features = 0.5 Max samples = 0.5 Min samples leaf = 5 Min samples split = 2 Initial features = 179 Features selected = 31 | C-index (mean 5-fold cross validation) = 72.53% C-index (test data) = 69.41% | Max depth = 50 Max features = 0.2 Min samples leaf = 5 Min samples split = 2 Subsample = 0.7 Initial features = 179 Features selected = 14 | C-index (mean 5-fold cross validation) = 72.11% C-index (test data) = 69.62% |  |  |
| **2^nd^ set** | L1 ratio = 1 Alpha = 0.0099 Initial features = 179 Features selected = 18 | C-index (mean 5-fold cross validation) = 71.71% C-index (test data) = 71.34% | Max depth = 50 Max features = 0.5 Max samples = 0.5 Min samples leaf = 5 Min samples split = 2 Initial features = 179 Features selected = 31 | C-index (mean 5-fold cross validation) = 72.51% C-index (test data) = 69.38% | Max depth = 20 Max features = 0.2 Min samples leaf = 1 Min samples split = 2 Subsample = 0.7 Initial features = 179 Features selected = 14 | C-index (mean 5-fold cross validation) = 72.24% C-index (test data) = 69.38% | Num nodes = 64 Dropout = 0.69 Learning rate = 0.010 Batch size = 256 Number of epochs = 120 Initial features = 179 Features selected = 42 | C-index (mean 5-fold cross validation) = 72.11% C-index (test data) = 68.84% |
| **3^rd^ set** | L1 ratio = 1 Alpha = 0.0099 Initial features = 148 Features selected = 25 | C-index (mean 5-fold cross validation) = 71.70% C-index (test data) = 70.41% | Max depth = 20 Max features = 0.5 Max samples = 0.5 Min samples leaf = 3 Min samples split = 10 Initial features = 149 Features selected = 28 | C-index (mean 5-fold cross validation) = 72.49% C-index (test data) = 69.78% | Max depth = 20 Max features = 0.2 Min samples leaf = 3 Min samples split = 10 Subsample = 0.5 Initial features = 149 Features selected = 11 | C-index (mean 5-fold cross validation) = 71.89% C-index (test data) = 69.05% |  |  |
| **4^th^ set** | **L1 ratio = 1 Alpha = 0.0099 Initial features = 149 Features selected = 18** | **C-index (mean 5-fold cross validation) = 71.74% C-index (test data) = 71.34%** | Max depth = 20 Max features = 0.5 Max samples = 0.5 Min samples leaf = 3 Min samples split = 10 Initial features = 149 Features selected = 28 | C-index (mean 5-fold cross validation) = 72.48% C-index (test data) = 69.76% | Max depth = 20 Max features = 0.2 Min samples leaf = 1 Min samples split = 6 Subsample = 0.5 Initial features = 149 Features selected = 11 | C-index (mean 5-fold cross validation) = 71.85% C-index (test data) = 68.76% | Num nodes = 128 Dropout = 0.68 Learning rate = 0.014 Batch size = 256 Number of epochs = 186 Initial features = 149 Features selected = 39 | C-index (mean 5-fold cross validation) = 71.97% C-index (test data) = 70.03% |

**Colorectal cancer**

| **Dataset** | **Cox Elastic Net regression** | | **Random Survival Forest** | | **Gradient Boosting Survival** | | **DeepSurv Neural Network** | |
| --- | --- | --- | --- | --- | --- | --- | --- | --- |
| **1^st^ set** | L1 ratio = 0.4 Alpha = 0.0180 Initial features = 426 Features selected = 42 | C-index (mean 5-fold cross validation) = 71.61% C-index (test data) = 70.16% | Max depth = 20 Max features = 0.2 Max samples = 0.7 Min samples leaf = 2  Min samples split = 10 Initial features = 426 Features selected = 61 | C-index (mean 5-fold cross validation) = 72.33% C-index (test data) = 69.88% | Max depth = 50 Max features = 0.2 Min samples leaf = 3 Min samples split = 2 Subsample = 0.7 Initial features = 426 Features selected = 31 | C-index (mean 5-fold cross validation) = 72.10% C-index (test data) = 68.53% |  |  |
| **2^nd^ set** | L1 ratio = 0.5 Alpha = 0.0110 Initial features = 426 Features selected = 44 | C-index (mean 5-fold cross validation) = 71.36% C-index (test data) = 69.97% | Max depth = 20 Max features = 0.2 Max samples = 0.7 Min samples leaf = 3 Min samples split = 10 Initial features = 426 Features selected = 61 | C-index (mean 5-fold cross validation) = 72.31% C-index (test data) = 69.90% | Max depth = 20 Max features = 0.2 Min samples leaf = 3 Min samples split = 2 Subsample = 0.7 Initial features = 426 Features selected = 31 | C-index (mean 5-fold cross validation) = 72.10% C-index (test data) = 68.74% | Num nodes = 64 Dropout = 0.53 Learning rate = 0.012 Batch size = 256 Number of epochs = 131 Initial features = 428 Features selected = 88 | C-index (mean 5-fold cross validation) = 74.39% C-index (test data) = 66.98% |
| **3^rd^ set** | **L1 ratio = 0.4 Alpha = 0.0150 Initial features = 138 Features selected = 39** | **C-index (mean 5-fold cross validation) = 71.70% C-index (test data) = 70.20%** | Max depth = 50 Max features = 0.2 Max samples = 0.5 Min samples leaf = 5 Min samples split = 2 Initial features = 138 Features selected = 28 | C-index (mean 5-fold cross validation) = 71.94% C-index (test data) = 69.76% | Max depth = 50 Max features = 0.2 Min samples leaf = 5 Min samples split = 2 Subsample = 0.5 Initial features = 138 Features selected = 16 | C-index (mean 5-fold cross validation) = 71.95% C-index (test data) = 69.15% |  |  |
| **4^th^ set** | L1 ratio = 0.7 Alpha = 0.0083 Initial features = 138 Features selected = 31 | C-index (mean 5-fold cross validation) = 71.48% C-index (test data) = 70.16% | Max depth = 50 Max features = 0.2 Max samples = 0.5 Min samples leaf = 5 Min samples split = 2 Initial features = 138 Features selected = 28 | C-index (mean 5-fold cross validation) = 71.94% C-index (test data) = 69.72% | Max depth = 20 Max features = 0.2 Min samples leaf = 1 Min samples split = 10 Subsample = 0.7 Initial features = 138 Features selected = 16 | C-index (mean 5-fold cross validation) = 71.83% C-index (test data) = 68.60% | Num nodes = 32 Dropout = 0.69 Learning rate = 0.036 Batch size = 64 Number of epochs = 183 Initial features = 138 Features selected = 31 | C-index (mean 5-fold cross validation) = 70.46% C-index (test data) = 68.55% |

**Endometrial cancer**

| **Dataset** | **Cox Elastic Net regression** | | **Random Survival Forest** | | **Gradient Boosting Survival** | | **DeepSurv Neural Network** | |
| --- | --- | --- | --- | --- | --- | --- | --- | --- |
| **1^st^ set** | **L1 ratio = 1 Alpha = 0.0168 Initial features = 238 Features selected = 25** | **C-index (mean 5-fold cross validation) = 73.17% C-index (test data) = 72.84%** | Max depth = 20 Max features = 0.2 Max samples = 0.7 Min samples leaf = 3 Min samples split = 2 Initial features = 238 Features selected = 37 | C-index (mean 5-fold cross validation) = 74.38% C-index (test data) = 72.65% | Max depth = 20 Max features = 0.8 Min samples leaf = 3 Min samples split = 10 Subsample = 0.5 Initial features = 238 Features selected = 27 | C-index (mean 5-fold cross validation) = 74.28% C-index (test data) = 71.27% |  |  |
| **2^nd^ set** | L1 ratio = 0.3 Alpha = 0.0847 Initial features = 238 Features selected = 7 | C-index (mean 5-fold cross validation) = 73.86% C-index (test data) = 71.95% | Max depth = 20 Max features = 0.2 Max samples = 0.7 Min samples leaf = 3 Min samples split = 2 Initial features = 238 Features selected = 37 | C-index (mean 5-fold cross validation) = 74.43% C-index (test data) = 72.67% | Max depth = 50 Max features = 0.2 Min samples leaf = 3 Min samples split = 2 Subsample = 0.7 Initial features = 238 Features selected = 27 | C-index (mean 5-fold cross validation) = 74.37% C-index (test data) = 71.53% | Num nodes = 64 Dropout = 0.52 Learning rate = 0.010 Batch size = 256 Number of epochs = 179 Initial features = 238 Features selected = 61 | C-index (mean 5-fold cross validation) = 83.49% C-index (test data) = 70.97% |
| **3^rd^ set** | L1 ratio = 1 Alpha = 0.0168 Initial features = 99 Features selected = 22 | C-index (mean 5-fold cross validation) = 73.24% C-index (test data) = 72.83% | Max depth = 20 Max features = 0.5 Max samples = 0.7 Min samples leaf = 3 Min samples split = 10 Initial features = 99 Features selected = 19 | C-index (mean 5-fold cross validation) = 73.81% C-index (test data) = 72.02% | Max depth = 20 Max features = 0.2 Min samples leaf = 1 Min samples split = 10 Subsample = 1.0 Initial features = 99 Features selected = 14 | C-index (mean 5-fold cross validation) = 73.75% C-index (test data) = 68.82% |  |  |
| **4^th^ set** | L1 ratio = 0.3 Alpha = 0.0641 Initial features = 99 Features selected = 10 | C-index (mean 5-fold cross validation) = 73.84% C-index (test data) = 72.21% | Max depth = 20 Max features = 0.5 Max samples = 0.7 Min samples leaf = 3 Min samples split = 10 Initial features = 99 Features selected = 19 | C-index (mean 5-fold cross validation) = 73.85% C-index (test data) = 71.98% | Max depth = 20 Max features = 0.2 Min samples leaf = 1 Min samples split = 10 Subsample = 1.0 Initial features = 99 Features selected = 14 | C-index (mean 5-fold cross validation) = 73.72% C-index (test data) = 68.74% | Num nodes = 32 Dropout = 0.63 Learning rate = 0.010 Batch size = 256 Number of epochs = 169 Initial features = 99 Features selected = 28 | C-index (mean 5-fold cross validation) = 77.67% C-index (test data) = 72.17% |

**Glioma**

| **Dataset** | **Cox Elastic Net regression** | | **Random Survival Forest** | | **Gradient Boosting Survival** | | **DeepSurv Neural Network** | |
| --- | --- | --- | --- | --- | --- | --- | --- | --- |
| **1^st^ set** | L1 ratio = 0.2 Alpha = 0.1099 Initial features = 90 Features selected = 28 | C-index (mean 5-fold cross validation) = 71.85% C-index (test data) = 76.93% | Max depth = 20 Max features = 0.2 Max samples = 0.7 Min samples leaf = 3 Min samples split = 10 Initial features = 90 Features selected = 22 | C-index (mean 5-fold cross validation) = 74.73% C-index (test data) = 78.19% | **Max depth = 50 Max features = 0.5 Min samples leaf = 1 Min samples split = 2 Subsample = 0.7 Initial features = 90 Features selected = 17** | **C-index (mean 5-fold cross validation) = 76.81% C-index (test data) = 79.97%** |  |  |
| **2^nd^ set** | L1 ratio = 0.9 Alpha = 0.0104 Initial features = 90 Features selected = 42 | C-index (mean 5-fold cross validation) = 73.07% C-index (test data) = 77.06% | Max depth = 20 Max features = 0.2 Max samples = 0.7 Min samples leaf = 3 Min samples split = 10 Initial features = 90 Features selected = 22 | C-index (mean 5-fold cross validation) = 74.81% C-index (test data) = 78.02% | Max depth = 50 Max features = 0.2 Min samples leaf = 1 Min samples split = 6 Subsample = 1.0 Initial features = 90 Features selected = 17 | C-index (mean 5-fold cross validation) = 76.64% C-index (test data) = 79.36% | Num nodes = 64 Dropout = 0.58 Learning rate = 0.010 Batch size = 256 Number of epochs = 126 Initial features = 90 Features selected = 27 | C-index (mean 5-fold cross validation) = 80.37% C-index (test data) = 78.96% |
| **3^rd^ set** | L1 ratio = 0.2 Alpha = 0.1099 Initial features = 80 Features selected = 26 | C-index (mean 5-fold cross validation) = 70.84% C-index (test data) = 76.27% | Max depth = 20 Max features = 0.5 Max samples = None Min samples leaf = 5 Min samples split = 2 Initial features = 80 Features selected = 21 | C-index (mean 5-fold cross validation) = 74.84% C-index (test data) = 79.28% | Max depth = 20 Max features = 0.8 Min samples leaf = 3 Min samples split = 2 Subsample = 1.0 Initial features = 80 Features selected = 13 | C-index (mean 5-fold cross validation) = 75.37% C-index (test data) = 77.39% |  |  |
| **4^th^ set** | L1 ratio = 1 Alpha = 0.0165 Initial features = 80 Features selected = 21 | C-index (mean 5-fold cross validation) = 72.32% C-index (test data) = 75.55% | Max depth = 20 Max features = 0.8 Max samples = None Min samples leaf = 5 Min samples split = 2 Initial features = 80 Features selected = 21 | C-index (mean 5-fold cross validation) = 74.90% C-index (test data) = 78.10% | Max depth = 20 Max features = 0.5 Min samples leaf = 1 Min samples split = 2 Subsample = 0.7 Initial features = 80 Features selected = 13 | C-index (mean 5-fold cross validation) = 75.58% C-index (test data) = 77.72% | Num nodes = 128 Dropout = 0.52 Learning rate = 0.013 Batch size = 256 Number of epochs = 104 Initial features = 80 Features selected = 22 | C-index (mean 5-fold cross validation) = 77.62% C-index (test data) = 74.66% |

**Leukaemia**

| **Dataset** | **Cox Elastic Net regression** | | **Random Survival Forest** | | **Gradient Boosting Survival** | | **DeepSurv Neural Network** | |
| --- | --- | --- | --- | --- | --- | --- | --- | --- |
| **1^st^ set** | L1 ratio = 1 Alpha = 0.0791 Initial features = 76 Features selected = 10 | C-index (mean 5-fold cross validation) = 71.19% C-index (test data) = 69.08% | Max depth = 20 Max features = 0.5 Max samples = 0.7 Min samples leaf = 3 Min samples split = 2 Initial features = 77 Features selected = 18 | C-index (mean 5-fold cross validation) = 71.27% C-index (test data) = 69.19% | Max depth = 20 Max features = 0.5 Min samples leaf = 1 Min samples split = 6 Subsample = 0.5 Initial features = 77 Features selected = 16 | C-index (mean 5-fold cross validation) = 71.28% C-index (test data) = 74.95% |  |  |
| **2^nd^ set** | L1 ratio = 0.1 Alpha = 0.1394 Initial features = 77 Features selected = 42 | C-index (mean 5-fold cross validation) = 70.33% C-index (test data) = 72.71% | Max depth = 20 Max features = 0.5 Max samples = 0.7 Min samples leaf = 3 Min samples split = 2 Initial features = 77 Features selected = 18 | C-index (mean 5-fold cross validation) = 71.24% C-index (test data) = 68.66% | Max depth = 20 Max features = 0.2 Min samples leaf = 1 Min samples split = 10 Subsample = 0.5 Initial features = 77 Features selected = 16 | C-index (mean 5-fold cross validation) = 71.09% C-index (test data) = 74.09% | Num nodes = 128 Dropout = 0.57 Learning rate = 0.011 Batch size = 256 Number of epochs = 125 Initial features = 77 Features selected = 33 | C-index (mean 5-fold cross validation) = 86.24% C-index (test data) = 68.71% |
| **3^rd^ set** | L1 ratio = 1 Alpha = 0.0791 Initial features = 70 Features selected = 10 | C-index (mean 5-fold cross validation) = 71.19% C-index (test data) = 69.08% | Max depth = 20 Max features = 0.5 Max samples = 0.7 Min samples leaf = 3 Min samples split = 2 Initial features = 71 Features selected = 17 | C-index (mean 5-fold cross validation) = 71.48% C-index (test data) = 70.47% | **Max depth = 20 Max features = 0.5 Min samples leaf = 1 Min samples split = 6 Subsample = 0.5 Initial features = 71 Features selected = 16** | **C-index (mean 5-fold cross validation) = 71.16% C-index (test data) = 76.23%** |  |  |
| **4^th^ set** | L1 ratio = 0.1 Alpha = 0.1157 Initial features = 71 Features selected = 42 | C-index (mean 5-fold cross validation) = 70.55% C-index (test data) = 72.49% | Max depth = 20 Max features = 0.5 Max samples = 0.7 Min samples leaf = 1 Min samples split = 10 Initial features = 71 Features selected = 17 | C-index (mean 5-fold cross validation) = 71.51% C-index (test data) = 67.27% | Max depth = 20 Max features = 0.5 Min samples leaf = 1 Min samples split = 6 Subsample = 0.5 Initial features = 71 Features selected = 16 | C-index (mean 5-fold cross validation) = 71.07% C-index (test data) = 75.69% | Num nodes = 128 Dropout = 0.61 Learning rate = 0.012 Batch size = 256 Number of epochs = 156 Initial features = 71 Features selected = 28 | C-index (mean 5-fold cross validation) = 83.29% C-index (test data) = 67.80% |

**Lung cancer**

| **Dataset** | **Cox Elastic Net regression** | | **Random Survival Forest** | | **Gradient Boosting Survival** | | **DeepSurv Neural Network** | |
| --- | --- | --- | --- | --- | --- | --- | --- | --- |
| **1^st^ set** | L1 ratio = 0.5 Alpha = 0.0171 Initial features = 149 Features selected = 41 | C-index (mean 5-fold cross validation) = 66.64% C-index (test data) = 66.82% | Max depth = 50 Max features = 0.2 Max samples = None Min samples leaf = 3 Min samples split = 2 Initial features = 149 Features selected = 32 | C-index (mean 5-fold cross validation) = 67.07% C-index (test data) = 66.67% | Max depth = 50 Max features = 0.5 Min samples leaf = 1 Min samples split = 2 Subsample = 1.0 Initial features = 149 Features selected = 23 | C-index (mean 5-fold cross validation) = 67.78% C-index (test data) = 64.96% |  |  |
| **2^nd^ set** | L1 ratio = 1 Alpha = 0.0034 Initial features = 149 Features selected = 76 | C-index (mean 5-fold cross validation) = 66.65% C-index (test data) = 66.73% | Max depth = 50 Max features = 0.2 Max samples = None Min samples leaf = 3 Min samples split = 2 Initial features = 149 Features selected = 32 | C-index (mean 5-fold cross validation) = 67.07% C-index (test data) = 66.70% | Max depth = 50 Max features = 0.5 Min samples leaf = 1 Min samples split = 2 Subsample = 1.0 Initial features = 149 Features selected = 23 | C-index (mean 5-fold cross validation) = 67.76% C-index (test data) = 65.02% | Num nodes = 64 Dropout = 0.51 Learning rate = 0.011 Batch size = 256 Number of epochs = 166 Initial features = 149 Features selected = 43 | C-index (mean 5-fold cross validation) = 72.32% C-index (test data) = 63.26% |
| **3^rd^ set** | L1 ratio = 0.1 Alpha = 0.0360 Initial features = 131 Features selected = 77 | C-index (mean 5-fold cross validation) = 66.68% C-index (test data) = 66.84% | Max depth = 20 Max features = 0.8 Max samples = 0.5 Min samples leaf = 5 Min samples split = 2 Initial features = 131 Features selected = 29 | C-index (mean 5-fold cross validation) = 67.24% C-index (test data) = 66.46% | Max depth = 20 Max features = 0.2 Min samples leaf = 3 Min samples split = 2 Subsample = 1.0 Initial features = 131 Features selected = 23 | C-index (mean 5-fold cross validation) = 67.83% C-index (test data) = 65.95% |  |  |
| **4^th^ set** | **L1 ratio = 0.9 Alpha = 0.0034 Initial features = 131 Features selected = 79** | **C-index (mean 5-fold cross validation) = 66.73% C-index (test data) = 66.98%** | Max depth = 20 Max features = 0.8 Max samples = 0.5 Min samples leaf = 5 Min samples split = 2 Initial features = 131 Features selected = 29 | C-index (mean 5-fold cross validation) = 67.21% C-index (test data) = 66.51% | Max depth = 50 Max features = 0.2 Min samples leaf = 3 Min samples split = 10 Subsample = 0.7 Initial features = 131 Features selected = 23 | C-index (mean 5-fold cross validation) = 68.03% C-index (test data) = 66.31% | Num nodes = 64 Dropout = 0.56 Learning rate = 0.010 Batch size = 256 Number of epochs = 109 Initial features = 131 Features selected = 39 | C-index (mean 5-fold cross validation) = 71.74% C-index (test data) = 63.46% |

**Ovarian cancer**

| **Dataset** | **Cox Elastic Net regression** | | **Random Survival Forest** | | **Gradient Boosting Survival** | | **DeepSurv Neural Network** | |
| --- | --- | --- | --- | --- | --- | --- | --- | --- |
| **1^st^ set** | L1 ratio = 0.8 Alpha = 0.0302 Initial features = 97 Features selected = 22 | C-index (mean 5-fold cross validation) = 68.97% C-index (test data) = 69.13% | **Max depth = 20 Max features = 0.2 Max samples = None Min samples leaf = 5 Min samples split = 2 Initial features = 98 Features selected = 20** | **C-index (mean 5-fold cross validation) = 71.13% C-index (test data) = 71.56%** | Max depth = 20 Max features = 0.2 Min samples leaf = 5 Min samples split = 2 Subsample = 1.0 Initial features = 98 Features selected = 18 | C-index (mean 5-fold cross validation) = 72.55% C-index (test data) = 68.71% |  |  |
| **2^nd^ set** | L1 ratio = 0.9 Alpha = 0.0413 Initial features = 98 Features selected = 7 | C-index (mean 5-fold cross validation) = 69.90% C-index (test data) = 68.25% | Max depth = 20 Max features = 0.2 Max samples = None Min samples leaf = 5 Min samples split = 2 Initial features = 98 Features selected = 20 | C-index (mean 5-fold cross validation) = 71.07% C-index (test data) = 71.54% | Max depth = 20 Max features = 0.2 Min samples leaf = 5 Min samples split = 2 Subsample = 1.0 Initial features = 98 Features selected = 18 | C-index (mean 5-fold cross validation) = 72.43% C-index (test data) = 68.71% | Num nodes = 128 Dropout = 0.54 Learning rate = 0.010 Batch size = 256 Number of epochs = 124 Initial features = 98 Features selected = 33 | C-index (mean 5-fold cross validation) = 77.67% C-index (test data) = 63.68% |
| **3^rd^ set** | L1 ratio = 0.5 Alpha = 0.0131 Initial features = 87 Features selected = 58 | C-index (mean 5-fold cross validation) = 69.46% C-index (test data) = 66.97% | Max depth = 20 Max features = 0.2 Max samples = 0.7 Min samples leaf = 3 Min samples split = 2 Initial features = 88 Features selected = 18 | C-index (mean 5-fold cross validation) = 71.12% C-index (test data) = 69.72% | Max depth = 20 Max features = 0.5 Min samples leaf = 3 Min samples split = 2 Subsample = 0.5 Initial features = 88 Features selected = 15 | C-index (mean 5-fold cross validation) = 72.33% C-index (test data) = 64.65% |  |  |
| **4^th^ set** | L1 ratio = 0.9 Alpha = 0.0413 Initial features = 88 Features selected = 6 | C-index (mean 5-fold cross validation) = 69.95% C-index (test data) = 68.05% | Max depth = 20 Max features = 0.2 Max samples = 0.7 Min samples leaf = 3 Min samples split = 2 Initial features = 88 Features selected = 18 | C-index (mean 5-fold cross validation) = 71.15% C-index (test data) = 69.80% | Max depth = 20 Max features = 0.8 Min samples leaf = 3 Min samples split = 10 Subsample = 0.5 Initial features = 88 Features selected = 15 | C-index (mean 5-fold cross validation) = 71.64% C-index (test data) = 64.71% | Num nodes = 64 Dropout = 0.53 Learning rate = 0.011 Batch size = 256 Number of epochs = 137 Initial features = 88 Features selected = 28 | C-index (mean 5-fold cross validation) = 76.23% C-index (test data) = 62.37% |

**Prostate cancer**

| **Dataset** | **Cox Elastic Net regression** | | **Random Survival Forest** | | **Gradient Boosting Survival** | | **DeepSurv Neural Network** | |
| --- | --- | --- | --- | --- | --- | --- | --- | --- |
| **1^st^ set** | L1 ratio = 0.8 Alpha = 0.0302 Initial features = 97 Features selected = 22 | C-index (mean 5-fold cross validation) = 68.97% C-index (test data) = 69.13% | **Max depth = 20 Max features = 0.2 Max samples = None Min samples leaf = 5 Min samples split = 2 Initial features = 98 Features selected = 20** | **C-index (mean 5-fold cross validation) = 71.13% C-index (test data) = 71.56%** | Max depth = 20 Max features = 0.2 Min samples leaf = 5 Min samples split = 2 Subsample = 1.0 Initial features = 98 Features selected = 18 | C-index (mean 5-fold cross validation) = 72.55% C-index (test data) = 68.71% |  |  |
| **2^nd^ set** | L1 ratio = 0.9 Alpha = 0.0413 Initial features = 98 Features selected = 7 | C-index (mean 5-fold cross validation) = 69.90% C-index (test data) = 68.25% | Max depth = 20 Max features = 0.2 Max samples = None Min samples leaf = 5 Min samples split = 2 Initial features = 98 Features selected = 20 | C-index (mean 5-fold cross validation) = 71.07% C-index (test data) = 71.54% | Max depth = 20 Max features = 0.2 Min samples leaf = 5 Min samples split = 2 Subsample = 1.0 Initial features = 98 Features selected = 18 | C-index (mean 5-fold cross validation) = 72.43% C-index (test data) = 68.71% | Num nodes = 128 Dropout = 0.54 Learning rate = 0.010 Batch size = 256 Number of epochs = 124 Initial features = 98 Features selected = 33 | C-index (mean 5-fold cross validation) = 77.67% C-index (test data) = 63.68% |
| **3^rd^ set** | L1 ratio = 0.5 Alpha = 0.0131 Initial features = 87 Features selected = 58 | C-index (mean 5-fold cross validation) = 69.46% C-index (test data) = 66.97% | Max depth = 20 Max features = 0.2 Max samples = 0.7 Min samples leaf = 3 Min samples split = 2 Initial features = 88 Features selected = 18 | C-index (mean 5-fold cross validation) = 71.12% C-index (test data) = 69.72% | Max depth = 20 Max features = 0.5 Min samples leaf = 3 Min samples split = 2 Subsample = 0.5 Initial features = 88 Features selected = 15 | C-index (mean 5-fold cross validation) = 72.33% C-index (test data) = 64.65% |  |  |
| **4^th^ set** | L1 ratio = 0.9 Alpha = 0.0413 Initial features = 88 Features selected = 6 | C-index (mean 5-fold cross validation) = 69.95% C-index (test data) = 68.05% | Max depth = 20 Max features = 0.2 Max samples = 0.7 Min samples leaf = 3 Min samples split = 2 Initial features = 88 Features selected = 18 | C-index (mean 5-fold cross validation) = 71.15% C-index (test data) = 69.80% | Max depth = 20 Max features = 0.8 Min samples leaf = 3 Min samples split = 10 Subsample = 0.5 Initial features = 88 Features selected = 15 | C-index (mean 5-fold cross validation) = 71.64% C-index (test data) = 64.71% | Num nodes = 64 Dropout = 0.53 Learning rate = 0.011 Batch size = 256 Number of epochs = 137 Initial features = 88 Features selected = 28 | C-index (mean 5-fold cross validation) = 76.23% C-index (test data) = 62.37% |

**Renal cancer**

| **Dataset** | **Cox Elastic Net regression** | | **Random Survival Forest** | | **Gradient Boosting Survival** | | **DeepSurv Neural Network** | |
| --- | --- | --- | --- | --- | --- | --- | --- | --- |
| **1^st^ set** | L1 ratio = 0.1 Alpha = 0.0231 Initial features = 132 Features selected = 94 | C-index (mean 5-fold cross validation) = 72.04% C-index (test data) = 72.91% | Max depth = 20 Max features = 0.2 Max samples = 0.7 Min samples leaf = 1 Min samples split = 10 Initial features = 132 Features selected = 23 | C-index (mean 5-fold cross validation) = 72.80% C-index (test data) = 73.50% | Max depth = 50 Max features = 0.2 Min samples leaf = 1 Min samples split = 2 Subsample = 1.0 Initial features = 132 Features selected = 17 | C-index (mean 5-fold cross validation) = 73.71% C-index (test data) = 72.71% |  |  |
| **2^nd^ set** | L1 ratio = 0.1 Alpha = 0.0772 Initial features = 132 Features selected = 35 | C-index (mean 5-fold cross validation) = 72.49% C-index (test data) = 72.26% | Max depth = 20 Max features = 0.2 Max samples = 0.7 Min samples leaf = 5 Min samples split = 2 Initial features = 132 Features selected = 22 | C-index (mean 5-fold cross validation) = 72.16% C-index (test data) = 73.57% | Max depth = 20 Max features = 0.5 Min samples leaf = 1 Min samples split = 2 Subsample = 0.7 Initial features = 132 Features selected = 17 | C-index (mean 5-fold cross validation) = 73.66% C-index (test data) = 73.69% | Num nodes = 128 Dropout = 0.53 Learning rate = 0.010 Batch size = 256 Number of epochs = 135 Initial features = 132 Features selected = 32 | C-index (mean 5-fold cross validation) = 79.17% C-index (test data) = 70.22% |
| **3^rd^ set** | L1 ratio = 0.1 Alpha = 0.0335 Initial features = 123 Features selected = 76 | C-index (mean 5-fold cross validation) = 72.24% C-index (test data) = 72.60% | Max depth = 20 Max features = 0.2 Max samples = 0.5 Min samples leaf = 5 Min samples split = 2 Initial features = 123 Features selected = 22 | C-index (mean 5-fold cross validation) = 72.79% C-index (test data) = 72.70% | Max depth = 50 Max features = 0.5 Min samples leaf = 1 Min samples split = 6 Subsample = 0.7 Initial features = 123 Features selected = 15 | C-index (mean 5-fold cross validation) = 73.62% C-index (test data) = 72.25% |  |  |
| **4^th^ set** | L1 ratio = 0.1 Alpha = 0.0772 Initial features = 123 Features selected = 33 | C-index (mean 5-fold cross validation) = 72.55% C-index (test data) = 72.19% | Max depth = 50 Max features = 0.5 Max samples = None Min samples leaf = 3 Min samples split = 10 Initial features = 123 Features selected = 22 | C-index (mean 5-fold cross validation) = 72.76% C-index (test data) = 73.10% | **Max depth = 50 Max features = 0.8 Min samples leaf = 3 Min samples split = 10 Subsample = 0.5 Initial features = 123 Features selected = 15** | **C-index (mean 5-fold cross validation) = 73.78% C-index (test data) = 73.72%** | Num nodes = 32 Dropout = 0.57 Learning rate = 0.010 Batch size = 256 Number of epochs = 188 Initial features = 123 Features selected = 25 | C-index (mean 5-fold cross validation) = 74.61% C-index (test data) = 71.87% |

**Supplementary File 3.** Beeswarm plots summarising feature importances

**Bladder cancer**

**Breast cancer**

**Colorectal cancer**

**Endometrial cancer**

**Glioma**

**Leukaemia**

**Lung cancer**

**Ovarian cancer**

**Prostate cancer**

**Renal cancer**

**Supplementary File 4.** Waterfall charts for high and low risk patients as predicted by models

**Bladder cancer**

**Breast cancer**

**Colorectal cancer**

**Endometrial cancer**

**Glioma**

**Leukaemia**

**Lung cancer**

**Ovarian cancer**

**Prostate cancer**

**Renal cancer**
